# Nicotinamide Mononucleotide Ameliorates Sleep Deprivation‐Induced Gut Microbiota Dysbiosis and Restores Colonization Resistance against Intestinal Infections

**DOI:** 10.1002/advs.202207170

**Published:** 2023-01-25

**Authors:** Dan Fang, Tianqi Xu, Jingyi Sun, Jingru Shi, Fulei Li, Yanqing Yin, Zhiqiang Wang, Yuan Liu

**Affiliations:** ^1^ College of Veterinary Medicine Yangzhou University Yangzhou 225009 P. R. China; ^2^ Jiangsu Co‐innovation Center for Prevention and Control of Important Animal Infectious Diseases and Zoonoses Joint International Research Laboratory of Agriculture and Agri‐Product Safety the Ministry of Education of China Yangzhou University Yangzhou 225009 P. R. China; ^3^ Institute of Comparative Medicine Yangzhou University Yangzhou 225009 P. R. China

**Keywords:** colonization resistance, gut microbiota, intestinal infections, metabolites, sleep deprivation

## Abstract

Gut microbiota‐mediated colonization resistance (CR) is crucial in protecting the host from intestinal infections. Sleep deprivation (SD) is an important contributor in the disturbances of intestinal homeostasis. However, whether and how SD affects host CR remains largely unknown. Here, it is shown that SD impairs intestinal CR in mice, whereas nicotinamide mononucleotide (NMN) supplementation restores it. Microbial diversity and metabolomic analyses suggest that gut microbiota and metabolite profiles in SD‐treated mice are highly shaped, whereas NMN reprograms these differences. Specifically, the altered gut microbiota in SD mice further incurs the disorder of secondary bile acids pool accompanied by a decrease in deoxycholic acid (DCA). Conversely, NMN supplementation retakes the potential benefits of DCA, which is associated with specific gut microbiota involved in primary bile acids metabolic flux. In animal models of infection, DCA is effective in preventing and treating bacterial infections when used alone or in combination with antibiotics. Mechanistically, DCA alone disrupts membrane permeability and aggravates oxidative damage, thereby reducing intestinal pathogen burden. Meanwhile, exogenous DCA promotes antibiotic accumulation and destroys oxidant–antioxidant system, thus potentiating antibiotic efficacy. Overall, this work highlights the important roles of gut microbiota and bile acid metabolism in the maintenance of intestinal CR.

## Graphical abstract

This work reports that sleep deprivation impairs intestinal colonization resistance and disrupts gut microbiota homeostasis in mice. Conversely, nicotinamide mononucleotide supplementation counteracts the negative effects of sleep deprivation, leading to the reassignment of microbiota‐associated bile acids pool. Deoxycholic acid, an important secondary bile acid, exhibits potential antibacterial activity and synergistic effect with existing antibiotics against pathogens.

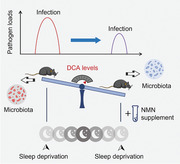

## 1 Introduction

Sleep occupies nearly a third of our lives and is of great importance in maintaining normal physiological activity in mammals. However, with the increasing pressure of work and the diversification of entertainment in modern society, sleep deprivation (SD) is becoming more frequent and common. Recently, the negative effects of SD on human health are being appreciated, including cardiovascular disease, the dysregulation of inflammatory responses, cognitive impairment, and systemic bacterial invasion.^[^ ^1^ ^]^ Furthermore, correlation networks between SD and gastrointestinal diseases have been an area of increasing interest during recent decades. Clinical studies have shown that sleep disorders contribute to the risk of inflammatory bowel disease infection (IBD),^[^ ^2^ ^]^ which has been listed as an important chronic inflammatory disease in healthcare.^[^ ^3^ ^]^ Even worse, SD leaves various detrimental effects on intestinal health, such as compromised gastric mucosal integrity, disturbed intestinal microbiota, and imbalanced microbiota metabolism.

Nowadays, intestinal infections caused by pathogens, especially multidrug‐resistant (MDR) bacteria, have been a critical risk factor that threatens human health. The global prevalence of antibiotic resistance and the cliff‐like reduction of new antibacterial drugs exacerbate the difficulty in the treatment of intestinal infections. In addition to being an anaerobic bioreactor programmed with an enormous population of bacteria, the intestinal lumen ultimately serves as a natural reservoir for antibiotic resistance genes (ARGs), in which plasmid‐mediated horizontal transfer of ARGs occurs,^[^ ^4^ ^]^ especially during antibiotic therapy.^[^ ^5^ ^]^ Therefore, it is imperative to develop safer and more reasonable strategies to counteract serious intestinal infections.

Modulating host colonization resistance (CR) may provide a feasible means for this purpose, which refers to the formation of a barrier by commensal microbiota to prevent the invasion of pathogenic bacteria.^[^ ^6^ ^]^ Accordingly, bacterial intestinal infection is closely related to the depletion of intestinal CR, which is directly or indirectly regulated by the gut microbiota and host activity. On the one hand, the contribution of gut microbiota to CR, while the detailed mechanisms are not fully resolved, can manifest in at least the following ways, including the secretion of antimicrobial products such as sulfide,^[^ ^6^ ^]^ forming competitive nutritional restriction and supporting gut barrier integrity.^[^ ^7^ ^]^ Dysbiosis of the gut microbiota and damage to the intestinal mucosa, in turn, can cause disturbances in circadian rhythms, lipid absorption and storage, and metabolic diseases including type 2 diabetes, nonalcoholic fat, and obesity.^[^ ^8^ ^]^ On the other hand, human activities such as a high‐fat diet (HFD) and antibiotic therapies, have been proved to confer a negative effect on the gut microbial community, thus impairing a wide range of functions it may provide. For example, the offspring exhibited impaired CR and showed more susceptibility to infectious colitis in the case of maternal intake of dietary fat,^[^ ^9^ ^]^ and antibiotic administration could collaterally alter the microbiota composition and lead to the loss of CR.^[^ ^10^ ^]^ Literally, these studies advance our understanding of the microbe–host interaction networks. However, the potential correlation between SD and intestinal infections, and the roles of gut microbiota in this process, remain elusive. Therefore, the aim of this study is to investigate the impact of SD on host CR and the following bacterial intestinal infections.

Nicotinamide mononucleotide (NMN), a product of the nicotinamide phosphoribosyl transferase reaction and a key NAD^+^ intermediate, was first discovered in 2011 and reported to ameliorate glucose intolerance by advancing NAD^+^ levels in HFD‐induced type 2 diabetes (T2D) mice.^[^ ^11^ ^]^ Moreover, NMN displays unique functions in promoting hepatic gluconeogenesis, insulin secretion, muscle insulin sensitivity, and adipose tissue lipogenesis.^[^ ^12^ ^]^ In particular, NMN treatment also contributes to the maintenance of intestinal health, including the integrity of the intestinal epithelium, the structure of the gut microbiota, and homeostasis of intestinal metabolism.^[^ ^13^ ^]^ Based on these, we simultaneously explored the potential beneficial effects of NMN in the control of intestinal infections.

## 2 Results

### 2.1 NMN Supplementation Alleviates SD‐Induced Depletion of Intestinal CR in Mice

**Figure 1:**
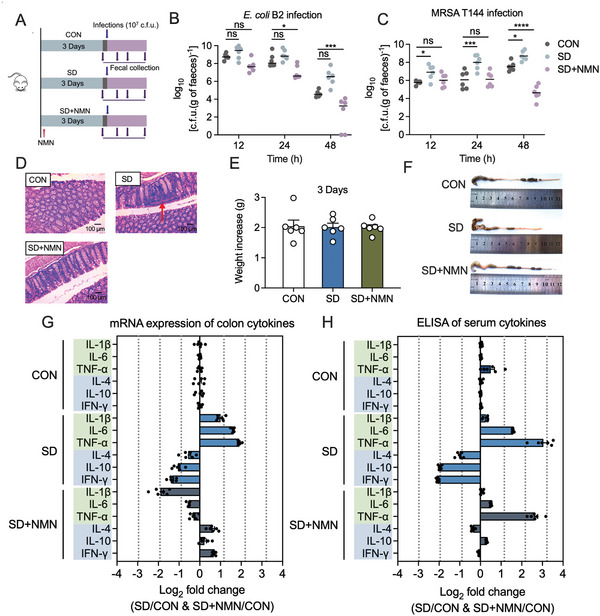
NMN supplementation rescues SD‐elicited depletion of intestinal CR in mice. A) Experimental protocols of SD‐induced intestinal infection model. The mice were randomly divided into three groups (*n* = 6 independent animals per group), including CON, SD, and SD + NMN groups. CON, healthy unperturbed group; SD, sleep‐deprived group; SD + NMN, sleep‐deprived and NMN‐supplied group (NMN: 100 mg kg^−1^ per day). B,C) Bacterial loads in mice feces at 12, 24, and 48 h post‐infection by B) *E. coli* B2 or C) MRSA T144. D) H&E staining of the colon tissues (original magnification: ×400). Inflammation aggravation of colon was observed in SD‐treated mice as indicated by the pathologic expansion of lymphocyte infiltration, marked by red arrows. Scar bar, 100 µm. E) Body weight changes of mice in CON, SD, and SD + NMN groups (*n* = 6 independent animals per group). F) Representative images of mice colon in three groups. Among them, colon atrophy and mucosal bleeding were observed in SD‐treated mice. G) RT‐qPCR analysis of inflammatory cytokines in colon samples of CON, SD, SD + NMN mice. H) ELISA analysis of inflammatory cytokines in serum samples of CON, SD, SD + NMN mice. In (G) and (H), fold changes between two groups were logarithmically compared. The CON group was used as a reference for normalization. Pro‐inflammatory cytokines (IL‐1*β*, IL‐6, TNF‐*α*) were marked with light green and anti‐inflammatory cytokines (IL‐4, IL‐10, INF‐*γ*) were marked with light blue. Statistical significance in (B) and (C) was assessed by two‐way ANOVA with Sidak's multiple comparison test. *p < 0.05, **p < 0.01, ***p < 0.001, ****p < 0.0001. ns, not significant. Statistical significance in (E) was determined by unpaired *t*‐test and denoted as follows: *p < 0.05, **p < 0.01, ***p < 0.001, ****p < 0.0001. ns, not significant.

To construct the SD mice model, we used a continuously rotating machine to interfere with the mice sleep for 3 days, and SD + NMN group mice were supplied with NMN via oral administration every day during the SD period (**Figure** **1**A). Results showed that there was no significant difference in weight gain between the three groups, implying that all mice had a synchronized diet during the experimental period (Figure 1E). Then we conducted bacterial enteric infection models by gavage administration with two representative pathogens, including MRSA T144 and *E. coli* B2. Interestingly, we found that SD‐treated group featured 10–1000‐fold higher fecal pathogens loads than the control (CON) group, as well as the inefficient eliminating capacity by 48 h postinfection. In contrast, SD + NMN mice obtained a remarkable decrease in both MRSA T144 and *E. coli* B2 loads compared with the SD‐treated group (Figure 1B,C). Consistently, colonic atrophy and mucosal congestion were observed in SD‐treated mice, whereas NMN supplementation attenuated these pathological processes inflicted by SD (Figure 1F). Furthermore, we assessed the intestinal inflammatory response in three groups via pathological features and the production of inflammatory factors in the host. In agreement with the results above, excessive infiltration of lymphocytes was observed in the SD‐treated group, whereas it was alleviated in SD + NMN group (Figure 1D). The real‐time quantitative reverse transcription polymerase chain reaction (RT‐qPCR) results showed that SD induced an approximately one to fourfold reduction of anti‐inflammatory cytokines (IL‐4, IL‐10, and INF‐*γ*) and an approximately one to eightfold increase of pro‐inflammatory cytokines (IL‐1*β*, IL‐6, and TNF‐*α*) both in the colon and liver compared with those of CON group. Conversely, NMN supplementation attenuated the stimulatory effect of SD on the changes in the cytokines mRNA expression (Figure 1G and Figure S1A,C, Supporting Information). Consistent with RT‐qPCR results, the concentrations of pro‐inflammatory cytokines (IL‐1*β*, IL‐6, and TNF‐*α*) in mice serum were dramatically increased in the SD‐treated group (one to eightfold), but the levels of anti‐inflammatory cytokines (IL‐4, IL‐10, and INF‐*γ*) were two to four times lower than those in CON group (Figure 1H and Figure S1B, Supporting Information). Totally, these data demonstrate that SD impairs intestinal CR, as well as the colon homeostasis in mice, while NMN supplementation indeed effectively reverses these changes.

### 2.2 Gut Microbiota and Bile Acids Metabolism are Highly Shaped in Response to SD While NMN Reprograms the Gut Flora

**Figure 2:**
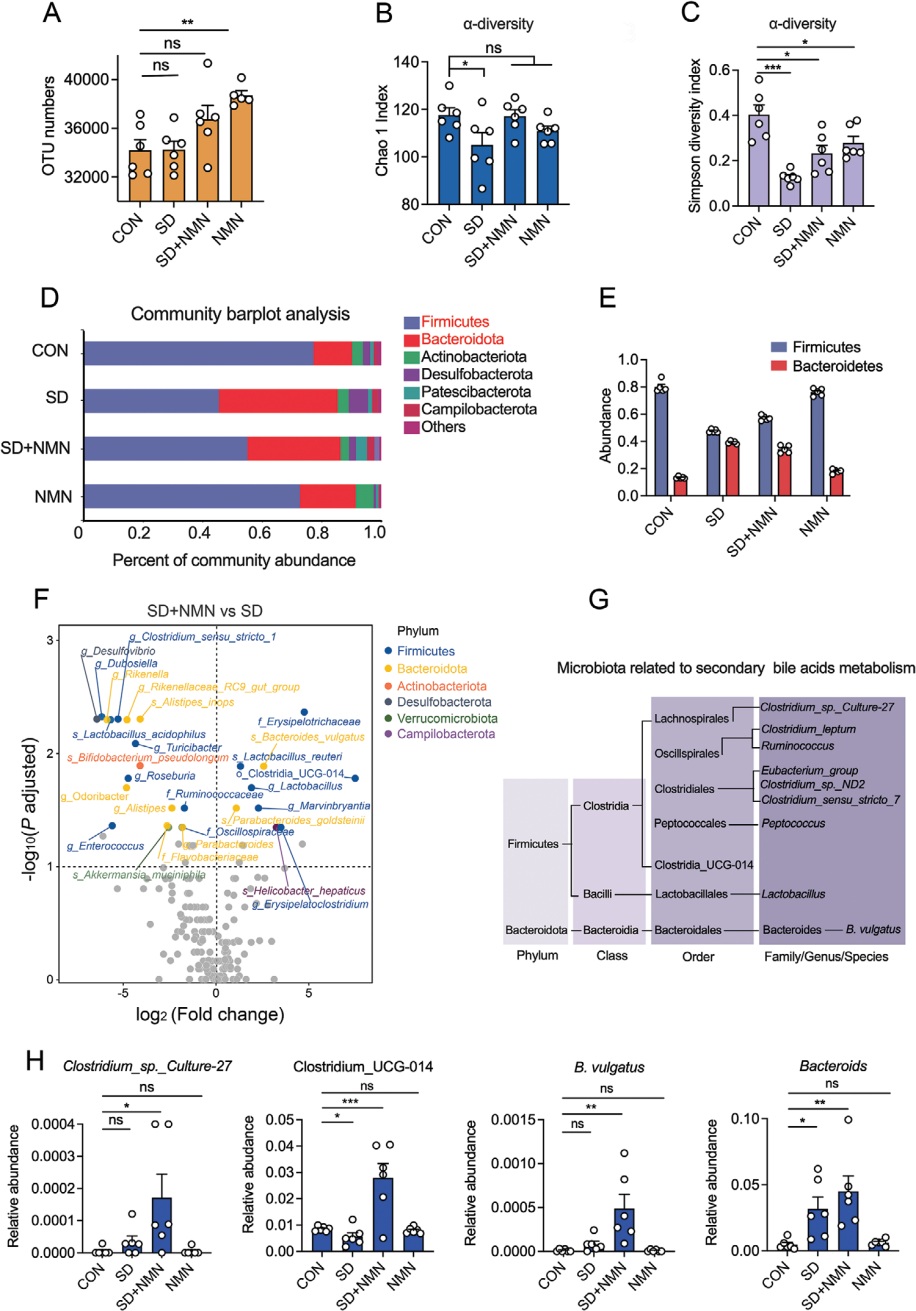
Microbial diversity analysis of gut microbiota between four groups. A) The number of OTUs in four groups. B,C) Alpha diversity of gut microbiota responses to NMN treatment in SD mice, including B) Chao 1 richness and C) Simpson diversity. Decreased Chao 1 abundance and Simpson diversity index in SD‐treated mice indicated the perturbations in gut microbiota diversity at the OTU level. D) Microbial population distribution in fecal samples at the phylum level. The horizontal axis represents the relative abundance of microbial population and the vertical axis represents samples’ information. E) The abundance of Firmicutes and Bacteroidetes in four groups. It shows a markedly lower ratio of Firmicutes to Bacteroidetes in SD‐treated mice, for the relative abundance of the Firmicutes in SD‐treated mice was lower by 50%, whereas the Bacteroidetes were higher by a corresponding degree. F) Volcano plots of differential bacteria associated with SD + NMN group versus SD group. Significantly up‐ or down‐regulated microbiota flora were colored according to their taxonomy at the phylum level. G) Taxonomic list of gut microbiota in our study that involved in bile acids metabolism, including primary bile acids metabolism and secondary bile acids metabolism. H) The relative abundance of *Clostridium_sp._Culture‐27*, Clostridium_UCG‐014, *Bacteroides vulgatus*, and *Bacteroids* among four groups. Data were presented as mean ± SEM. Statistical significance was determined by unpaired *t*‐test and denoted as follows: *p < 0.05; **p < 0.01; ***p < 0.001; ns, not significant.

Gut microbiota has been implicated in the maintenance of host CR and the prevention of invading pathogens.^[^ ^14^ ^]^ To explore the alternation of intestinal microbiota between four groups (the fourth group represented the mice treated with NMN alone to monitor the action of NMN), microbiota profiling was performed to analyze the diversity and composition of gut bacterial community. Microbial diversity results showed that NMN group had the most significant changes in the number of operational taxonomic units (OTUs, **Figure** **2**A), which was increased by 13.17% (*p* = 0.0046) compared with that in the CON group, however, no significant difference between SD and SD + NMN group was observed. Chao 1 and Simpson indexes were further applied for *α*‐diversity analysis of the gut microbiome. As a consequence, SD‐treated mice showed a lower richness and abundance of microbiota evidenced by the decreased Chao 1 and Simpson indexes compared to the CON group (Figure 2B,C). Interestingly, both SD + NMN and NMN groups displayed an approximate increase in Chao 1 and Simpson indexes compared with the SD‐treated group, suggesting that NMN supplementation resulted in an improvement in gut microbiota diversity. In addition, principal component analysis (PCA), one component of *β*‐diversity, implied that SD caused remarkable structural changes of fecal microbiota while SD + NMN largely restored these changes, since these SD + NMN labeled plots showed a trend of regression to the CON group from the SD group (Figure S2A, Supporting Information).

Microbial composition results in four groups were presented at the phylum level (Figure 2D), the two largest phyla were Firmicutes and Bacteroidetes. Furthermore, the ratio of Firmicutes to Bacteroidetes, one of the key indicators to reflect the homeostasis of gut microbial ecology,^[^ ^15^ ^]^ experienced a 78.23% decrease in the SD‐treated group, a 74.95% decrease in SD + NMN group, and a 29.98% decrease in NMN group compared with that in CON group (Figure 2E). In addition, distributions of the dominant bacterial population in four groups at the class level were visualized by Circos (Figure S2B, Supporting Information).^[^ ^16^ ^]^ The switch of the most dominant flora was centered on Lactobacillaceae and Muribaculaceae, namely, in CON, SD, SD + NMN groups, Lactobacillaceae accounted for 65%, 19%, 37%, respectively, and Muribaculaceae accounted for 11%, 20%, 24%, respectively, of the whole flora. Moreover, we conducted the LEfSe analysis to further clarify the differential microbial biomarkers. Key bacteria at different taxonomy levels are listed in Figure S2C,D in the Supporting Information (LDA > 2) by ways of testing both the consistency and effect size of the difference in bacteria abundance.^[^ ^17^ ^]^

Herein, we identified that 27 bacterial species were significantly reprogramed in SD + NMN mice (*p* adjusted value < 0.05), the vast majority of upregulated microbiota in SD + NMN mice were concentrated on highly abundant phyla, Firmicutes. Compared with SD mice, longitudinal comparisons of bacterial taxa disclosed the salient upregulation of o_Clostridia_UCG‐014, *g_Lactobacillus* and the remarkable downregulation of *g_desulfovibrio* in SD + NMN mice (Figure 2F). Interestingly, both these three bacterial taxa were reported to be associated with bile acids metabolism in colon in biological process, as *Clostridium* genera possess deconjugation, 7‐dehyfroxylation activity of primary bile acids and *desulfovibrio* is linked with cecal secondary bile acids production,^[^ ^18^ ^]^ suggesting that the composition of secondary bile acids pool may encounter a huge reassignment in SD and SD + NMN mice.

Gut microbiota‐mediated bile salt conversions in colon mainly involve deconjugation, epimerization, 7‐dehydroxylation, esterification, and desulfatation.^[^ ^19^ ^]^ Here, we enumerated the microbiota involved in these five sorts of conversions (Figure 2G) and analyzed their relative abundance in the four groups, respectively (Figure 2H and Figure S2E, Supporting Information). As shown in Figure 2H, the highest abundance of four bacteria (*Clostridium_sp._Culture‐27*, Clostridium_UCG‐014, *B. vulgatus*, and *Bacteroids*) was found in the SD + NMN group, implicating that they may play a potential role in the regulation of gut microbiota–bile acid–host axis and host CR. Taken together, our results reveal that SD has a negative regulatory effect on the diversity and structure of intestinal microorganisms, while NMN supplement mitigates SD‐induced gut microbiota dysbiosis.

### 2.3 Altered Gut Microbiota by NMN Supplementation Redresses Primary Bile Acids Metabolism and thus Increases DCA Levels

**Figure 3:**
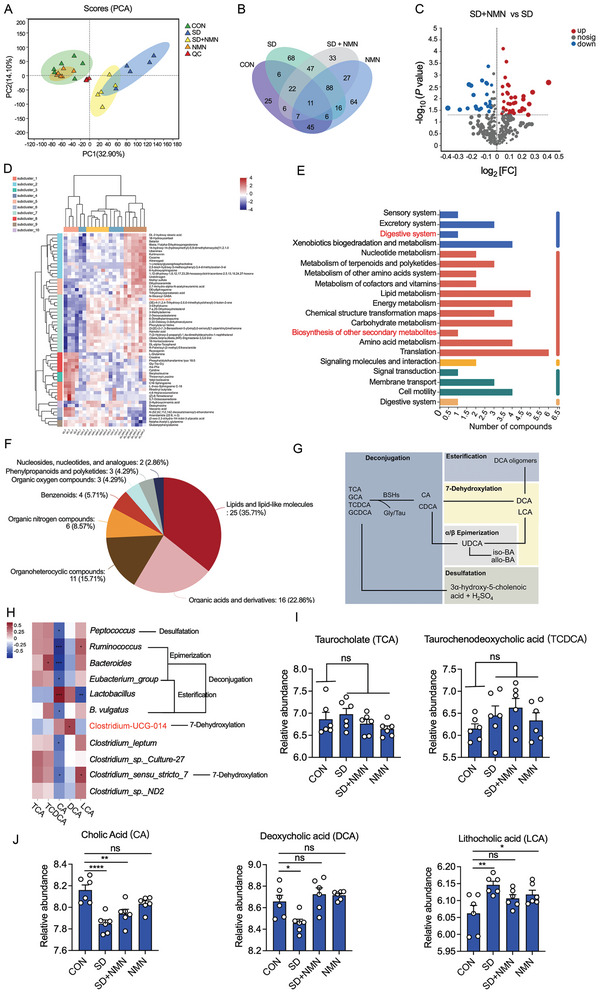
Alteration of mice fecal metabolite profiles among four groups. A) PCA analysis of identified metabolites in the feces from CON, SD, SD + NMN, NMN mice along with principal component (PC) 1 and 2, which explained 32.90%, 14.10% of the total variance, respectively. B) Venn diagrams showing the relationship of metabolites among four groups. C) Volcano map of differentially enriched metabolites in feces of SD + NMN mice compared with that in SD‐treated mice. Differentially enriched metabolites were identified by LC‐MS analysis with *p* values of ≤ 0.05 and FC values ≥ 2, and distinguished using one vertical and one horizontal dashed black line. Significantly up‐ and down‐regulated metabolites were shown in red and blue, respectively. Metabolites with no significant changes (nosing) in the gut were shown in gray. D) Heat‐map analyses of the top 50 metabolites in fecal samples from CON, SD, SD + NMN, NMN mice (*n* =  6 biologically independent animals per group). The color indicates the relative abundance of the metabolite in the group of samples, and the corresponding relationship between the color gradient and the values was shown in the gradient color block. DCA was labeled red. E) KEGG pathway enrichment analyses based on the significantly altered metabolites. The secondary classification category of KEGG compounds was shown on the left, and the number of metabolites annotated into this classification was shown on the right. The pathways for DCA involvement were marked in red. F) HMDB classifications of compounds with differential metabolites. Each color in the pie chart represented the different HMBD classifications, and its area represented the relative proportion of metabolites in the classification. The total numbers of significantly altered metabolites in this class were indicated and the corresponding proportions were shown in parentheses. G) The interconnection of the five main types of transformation of primary bile acids. H) Spearman correlation analyses between five bile acids metabolites and microbiota abundance that were related to bile acid metabolism. I,J) The relative abundance of I) conjugated bile acids (TCA and TCDCA) and J) free bile acids (CA) and secondary bile acids (DCA and LCA) in four groups. Fecal bile acids abundance was determined by gas chromatography. Data were presented as mean ± SEM. Statistical significance was assessed by unpaired *t*‐test and denoted as follows: *p < 0.05; **p < 0.01; ***p < 0.001; ns, not significant.

There is growing evidence that changes in metabolite abundance caused by gut microbiota dysbiosis are the main reasons for the depletion of CR.^[^ ^20^ ^]^ Thus, we proposed that the altered gut microbiota may activate potential metabolic pathways to confer the increased CR in SD + NMN group, which is accompanied by specific differential metabolites enrichment. To test this, we conducted untargeted metabolomics analyses of fecal examples collected from CON, SD, SD + NMN, and NMN groups. 2D PCA score plots demonstrated manifest separation in metabolite profiles among four groups, suggesting that the metabolome of feces in the CON group was largely different from that in the SD‐treated group (**Figure** **3**A). As shown in Figure 3B by Venn diagram, 168 of the 471 major differential metabolites were common in the four groups. 302 differential metabolites were detected between CON versus SD group and 169 differential metabolites were detected between SD versus SD + NMN group. Specifically, we found that SD + NMN led to 37 upregulated differentially enriched metabolites (DEMs) and 24 downregulated DEMs in SD + NMN group compared with that in the SD‐treated group (Figure 3C). The first top 60 metabolites, including 39 upregulated DEMs and 21 downregulated DEMs, are listed and clustered in Figure 3D. Then, we analyzed DEMs to their respective biochemical pathways as outlined in KEGG. Interestingly, the most enriched second category of KEGG pathways was amino acid metabolism, followed by the metabolism of cofactors and vitamins (Figure 3E). In addition, the first top 70 metabolites obtained from level‐two identification were assigned to the Human Metabolome Database (HMDB, Figure 3F), lipids and lipid‐like molecules were the first class (35.71%), followed by organic acids and derivatives (22.86%) and organoheterocyclic compounds (15.71%). Combined with microbial diversity analysis results, we further focused on metabolites associated with bile salts metabolism in the gut.

Generally, the free bile acids including cholic acid (CA) and chenodeoxycholic acid (CDCA) and their conjugated types comprise the primary bile acids pool. Through enterohepatic circulation, most of the primary bile acids are reabsorbed in the distal ileum and the remaining part will pass into the colon where they undergo bacterial conversions and lead to the enrichment of over 20 different secondary bile acids in feces.^[^ ^21^ ^]^ As shown in Figure 3G, the five main types of transformation of primary bile acids are interconnected, and deconjugation reaction mediated by bile salt hydrolases (BSHs) underlies the other four types of transformations. Furthermore, the most abundant and physiologically important metabolites of mammalian primary bile acids are deoxycholic acid (DCA) and lithocholic acid (LCA), which involve the conversion of CA to DCA by deconjugation and 7‐dehydroxylation, the conversation of CDCA to LCA by deconjugation, epimerization, and 7‐dehydroxylation, respectively.^[^ ^22^ ^]^ To further clarify the transformation relationship between specific bacteria and bile acids, we conducted a correlation analysis between 11 bacterial species and five bile acid‐related metabolites by Spearman correlation analysis (Figure 3H). Consequently, we found that DCA content was positively correlated to the relative abundance of Clostridium_UCG‐014, and LCA production was positively correlated to the relative abundance of *Ruminococcus* and *Clostridium_sensu_stricto_7* but negatively correlated to the relative abundance of *Lactobacillus*, suggesting that Clostridium_UCG‐014 may participate in the 7‐dehydroxylation of CA to DCA, while *Ruminococcus* and *Clostridium_sensu_stricto_7* may contribute to the production of LCA.

Next, we analyzed whether these specific gut microbiota‐mediated metabolic processes are associated with the reassignment of bile acid pool. In particular, the abundance of conjugated bile acids (TCA, TCDCA) in four groups exhibited no significant changes but deconjugated bile acid (CA) and secondary bile acids (DCA and LCA) changed to varying degrees (Figure 3I,J), hinting the reassignment of bile acids pool in colon and the differential transformational flux of secondary bile acids between SD and SD + NMN group, namely, LCA was dominant in SD group and DCA was dominant in SD + NMN group. Interestingly, the difference in bile acids in both groups coincided with that in gut microbiota, reasoning that the downregulated *Lactobacillus* in SD group may reduce the deconjugation of TCA or TCDCA to CA or CDCA, thus the lowest level of CA in feces was observed in SD mice. However, the upregulated *Ruminococcus* and *Clostridium_sensu_stricto_7* in SD group additionally multiplied the epimerization of CDCA to ursodeoxycholic acid (UDCA) and the 7‐dehydroxylation of UDCA to LCA, respectively, resulting in the highest level of LCA concentration (Figure S2E in the Supporting Information and Figure 3J). Conversely, upregulation of *Lactobacillus* and Clostridium_UCG‐014 in SD + NMN group actively incurred the deconjugation of TCA or GCA to CA and the 7‐dehydroxylation of CA to DCA (Figures 2H, 3J, and Figure S2E, Supporting Information). Together, these results suggest that SD gut microbiota‐mediated primary bile acids metabolism has two metabolic flux outcomes in the absence or presence of NMN supplementation, and the accumulated DCA may play an important role in maintaining host CR.

Additionally, NMN replenishment bolsters colonic DCA production can be conceptually understood by the driving of a considerable NAD^+^ pool after oral administration of NMN in SD mice. On the one hand, it has suggested that oral administration of NMN can be rapidly assimilated into the intestine with the assistance of specific transporter protein Slc12a8,^[^ ^23^ ^]^ followed by the consumption of adenosine triphosphate (ATP) to convert into NAD^+^, thus offering a high level of NAD^+^ availability in redox reactions that happened in these pathophysiological conditions. On the other hand, DCA is a well‐established bile acid 7‐dehydroxylation product by the gut microbiome and one of the major components of the recirculating pool of bile acids.^[^ ^24^ ^]^ It has proven that a set of six enzymes is necessary for the eight‐step reaction of CA to DCA in *Clostridium* spp. and the related pathways involve rapid NAD^+^ turnover.^[^ ^25^ ^]^ Hence, we conclude that the increase of the relative abundance of *Lactobacillus* and Clostridium_UCG‐014 along with exogenous NAD^+^ intermediates supplement may be an important reason accounting for the high levels of DCA in SD + NMN mice (Figure 7A).

### 2.4 DCA Exhibits Potent Antibacterial Activity as well as a Synergistic Effect with Antibiotics against Bacteria

**Figure 4:**
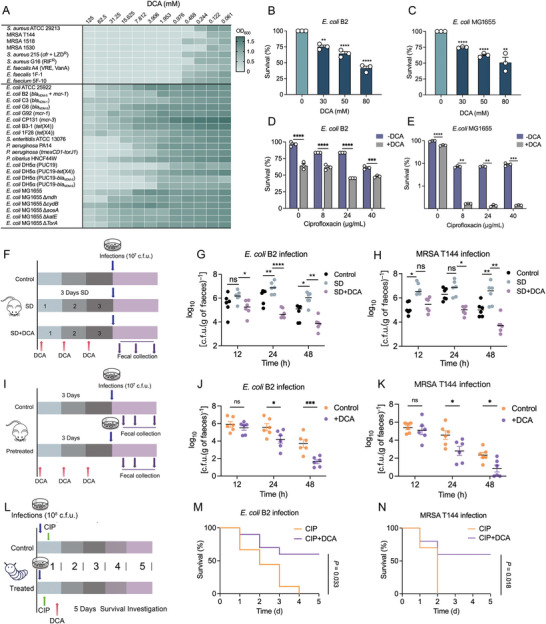
Antibacterial activity of DCA as well as its synergistic effect with existing antibiotics against bacterial infections. A) DCA sensitivity analysis in response to individual microbiota strains, including a panel of clinically drug‐resistant strains. Data represent the mean OD_600_ nm of three biological replicates. Dark green regions indicate higher cell density. B,C) Percent survival of B) *E. coli* B2 or C) *E. coli* MG1655 after exposure to increasing concentrations of DCA ranged from 0 × 10^−3^ to 80 × 10^−3^ m. D,E) DCA supplementation remarkably potentiates the bactericidal activity of CIP against D) multidrug‐resistant *E. coli* B2 and E) drug‐sensitive *E. coli* MG1655. F) Experimental protocols of DCA‐supplied intestinal infection model. The mice were randomly divided into three groups (*n* = 6 independent animals per group), including CON, SD, and SD + DCA groups. DCA administration, 100 mg kg^−1^ per day (i.p.). G,H) Bacterial loads in the feces of mice at 12, 24, and 48 h post‐infection by G) *E. coli* B2 or H) MRSA T144. I) Protocols of DCA‐pretreated administration in mice (*n* = 6 biologically independent animals per group). In the pretreated group, mice were supplied with a single intraperitoneal (i.p.) administration of DCA (100 mg kg^−1^) for 3 consecutive days. J,K) Fecal colonization of invading pathogens in DCA‐pretreated mice infected by J) *E. coli* B2 or K) MRSA T144 (10^7^ CFUs per mouse, i.g.). L) Protocol of the therapeutic potential assessment of combined use of DCA and CIP in vivo (*n* = 20 biologically independent larvae per group). M,N) Survival rate of *Galleria mellonella* larvae after 5 days postinfection by *E. coli* B2 or MRSA T144 (10^8^ CFUs per larvae) and treated by CIP (10 × 10^−6^ m kg^−1^) alone or in combination with DCA (20 × 10^−6^ m kg^−1^). Data in (B)–(N) were displayed as mean ± SEM, and statistical significance was determined by unpaired *t*‐test (*p < 0.05; **p < 0.01; ***p < 0.001; ns, not significant). Experiments were performed with three biological replicates. Statistical significance in (J) and (K) was assessed by two‐way ANOVA with Sidak's multiple comparison test.

Having shown that gut microbiota‐derived DCA may serve as a key metabolite in maintaining host CR, we next sought to explore its potential functions. We reasoned that the support of DCA for host CR may be due to its direct antibacterial effect. To verify this hypothesis, we assessed the antibacterial activity of DCA against a panel of bacteria, including reference strains and antibiotic‐resistant pathogens. As shown in **Figure** **4**A, DCA exhibited potent antimicrobial effects against Gram‐positive bacteria at a low concentration as well as slightly inferior activity against Gram‐negative bacteria across a concentration gradient ranging from 0.061 × 10^−3^ to 125  × 10^−3^ m. Considering that Gram‐negative pathogens have been the main cause of intestinal infections, we next focused on the antibacterial activity of DCA on two Gram‐negative strains, including MDR *E. coli* B2 and standard strain *E. coli* MG1655. Sterilization analysis indicated that the survival of two strains was significantly reduced under exposure of increasing concentrations of DCA (Figure 4B,C and Figure S3A,B, Supporting Information). Additionally, DCA showed similar bactericidal activity against *E. coli* DH5*α* and engineered *E. coli* DH5*α* carrying important antibiotic resistance genes (ARGs), including *bla*_NDM‐5_ or *tet*(X4) (Figure S3C,D, Supporting Information). These results indicate that the broad‐spectrum antimicrobial activity of DCA is independent of the existence of clinically important ARGs.

The drug combination approach represents a more cost‐effective strategy to combat drug‐resistant bacterial infections.^[^ ^26^ ^]^ Thus, we next evaluated the potentiating efficacy of DCA in combination with different classes of antibiotics against MDR *E. coli* B2. Excitingly, exogenous DCA supplementation markedly enhanced the bactericidal activity of multiple antibiotics including ciprofloxacin (CIP) (targeting DNA synthesis), kanamycin (targeting protein synthesis), and colistin (targeting membrane integrity) against *E. coli* B2 (Figure 4D and Figure S3E, Supporting Information). Besides, the synergistic activity of DCA with CIP against drug‐sensitive bacteria *E. coli* MG1655 can also be detected (Figure 4E), implying that the potentiating action of DCA was not dependent on the inhibition of specific resistance determinants.

Given the great antibacterial and synergistic activity of DCA in vitro, we further evaluated the efficacy of DCA in vivo. First, we found that 3‐day DCA administration enhanced CR against pathogens invasion in SD mice, including MRSA T144 and *E. coli* B2 infections (Figure 4F–H), indicating that DCA administration had the same benefits as NMN supplementation. Second, a mice model of infection was used to evaluate the preventive effect of DCA alone on bacterial infections. Mice were preadministrated with DCA for 3 continuous days and then infected with *E. coli* B2 or MRSA T144, bacterial loads in feces were determined at 12, 24, 48 h postinfection (Figure 4I). Surprisingly, the bacterial burden in DCA‐pretreated mice was significantly lower than that in the control group, as well as greater bacterial clearance efficiency (Figure 4J,K). Furthermore, *Galleria mellonella* infection models were applied to assess the therapeutic effects of DCA against bacterial infections. We found that a single dose of DCA (20  × 10^−6^ m kg^−1^) remarkably increased the survival rate of larvae, which was 40–70% higher than that in the control group (Figure S3F–H, Supporting Information). In addition, combined treatment of CIP and DCA resulted in more than 50% survival in the *E. coli* B2 and MRSA T144 infection models, whereas 100% of animals died within 4 days in the CIP‐alone‐treated group (Figure 4L–N). These results suggest that DCA may serve as a potent antimicrobial agent and antibiotic adjuvant to address various infections caused by MDR pathogens.

To explore whether only DCA plays a role in supporting host CR, we also evaluated the antibacterial activity of other metabolites that decreased in SD mice but increased in SD + NMN group (Figure S3I, Supporting Information). As shown in Figure S3J in the Supporting Information, only DCA inhibited *E. coli* B2 growth strikingly. In addition, LCA, as one of the two main secondary bile acids, has also been found to have a weak antibacterial effect.^[^ ^27^ ^]^ To verify the potential role of LCA, we assessed the effect of exogenous LCA supplementation on host CR in SD mice. As a consequence, we found that LCA exhibited little antibacterial activity compared with DCA (Figure S3K, Supporting Information). More intuitively, the administration of LCA to SD mice could not reverse the impaired CR in vivo (Figure S3L–N, Supporting Information). Overall, our results demonstrate that DCA content is of great importance in the maintenance of host CR, particularly in the context of SD‐induced intestinal impairment.

### 2.5 DCA Leads to the Increase of TCA Flux, ETC Activity, and Reactive Oxygen Species (ROS) Levels in *E. coli*

**Figure 5:**
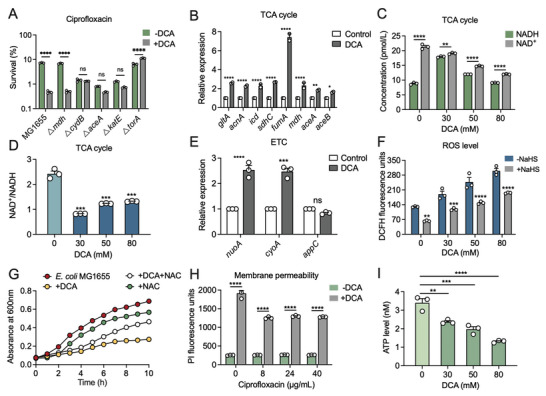
Antibacterial mechanisms of DCA against *E. coli*. A) Percent survival of *E. coli* MG1655 and related knockout strains (△*mdh*, △*cydB*, △*aceA*, △*katE*, and △*torA*) after 4 h co‐treatment of DCA (50 × 10^−3^ m) and CIP (20‐fold MIC). B) Effect of DCA (50 × 10^−3^ m) on the mRNA expression of enzymes involved in TCA cycles. C,D) Intracellular C) NAD^+^ and NADH levels, and D) the ratio of NAD^+^/NADH in *E. coli* MG1655 in the absence or presence of DCA (50 × 10^−3^ m). E) The mRNA expression levels of electron transport chain (ETC)‐related genes in *E. coli* MG1655 after exposure to DCA (50 × 10^−3^ m). F) ROS levels of *E. coli* MG1655 in response to the increasing concentration of DCA, and NaHS (30 × 10^−3^ m) was served as the direct H_2_S donor, which functions as the defense gas protecting bacteria from oxidative damage. G) Growth curves of *E. coli* MG1655 within 10 h in the presence of DCA with or without NAC (10 × 10^−3^ m), a ROS scavenger. H) Membrane permeability of *E. coli* MG1655 under the stimulation of varying concentrations of DCA for 1 h, probed by propidium iodide (10 × 10^−9^ m). I) Intracellular ATP levels of *E. coli* MG1655 in response to the increasing concentration of DCA ranged from 0 × 10^−3^ to 80 × 10^−3^ m. Data were displayed as mean ± SEM, and statistical significance was determined by unpaired *t*‐test (*p < 0.05; **p < 0.01; ***p < 0.001; ns, not significant). Experiments were performed with three biological replicates.

After confirming the antibacterial activity and synergistic action of DCA both in vitro and in vivo, we next set out to elucidate the underlying modes of action of DCA. Using the constructed mutants involving multilayered metabolic networks in our laboratory, we found that the synergistic effect of DCA was dramatically abolished in *E. coli* MG1655 Δ*cydB*, Δ*aceA*, and Δ*katE* strains, implying for the likely mechanisms of DCA were involved in the TCA cycle, electron respiration, and antioxidant system, respectively (**Figure** **5**A).

Considering that oxidative damage has been established as a common mechanism of cellular death,^[^ ^28^ ^]^ we proposed that DCA may act in a manner to ultimately induce oxidative damage, which results from a series of downstream lethality events on the involvement of the TCA cycle, cellular respiration, and altered metabolism. To verify our hypothesis, we first determined TCA cycle‐related biochemical changes in *E. coli* MG1655 treated by DCA. We found that the mRNA expression levels of TCA cycle‐related enzymes were remarkably upregulated in the presence of DCA (Figure 5B), and the NAD^+^/NADH ratio was reduced as a result of the increased NADH and the decreased NAD^+^ (Figure 5C,D). In principle, NADH is converted from NAD^+^ during the TCA cycle,^[^ ^28^ ^]^ therefore, these results suggested that the metabolic flux of the TCA cycle in *E. coli* MG1655 was increased by DCA. In addition, it has been acknowledged that oxygen and the conversion of NADH from NAD^+^ drive the oxidation of the respiratory electron transport chain (ETC) and thus produce more superoxide (O_2_^•−^) in *E. coli*.^[^ ^29^ ^]^ Consistently, we observed upregulated gene expression of ETC‐related enzymes in the presence of DCA, including *nuoA* and *cyoA* that encode quinone oxidoreductase subunit A and cytochrome bo3 ubiquinol oxidase subunit 2, respectively (Figure 5E). Therefore, we concluded that DCA considerably stimulated both TCA efflux and ETC activity. Moreover, accumulation of total ROS levels, including superoxide (O_2_^•−^), hydrogen peroxide (H_2_O_2_), and hydroxyl radical (HO•), can also be detected in DCA‐treated bacteria (Figure 5F). Conversely, the inhibition of bacterial growth induced by DCA could be reversed by the addition of *N*‐acetyl‐L‐cysteine (NAC), a scavenger of ROS, suggesting that ROS production played a vital role in the direct lethal effect of DCA on bacteria (Figure 5G). Cumulatively, these data demonstrate that the antibacterial activity of DCA is associated with metabolic perturbations in *E. coli* by stimulating TCA flux, enhancing ETC, and triggering the overproduction of ROS.

In addition, membrane permeability was remarkably disrupted by DCA and thus induced the disturbances of substances transportation, including protons, irons, etc. (Figure 5H), which also facilitated the final cell death. However, the intracellular ATP level was decreased in *E. coli* after exposure to DCA (Figure 5I) despite the enhanced ETC. On the basis of these results, we speculated that DCA, as a lipid‐soluble molecule, could first penetrate the phospholipid bilayer, then disrupt the formation of the proton gradient, eventually impairing the rotation of F_1_F_0_‐ATPase and the release of ATP.^[^ ^30^ ^]^ Together, the uncoupling of electron transport and oxidative phosphorylation may be one of the important reasons accounting for the enhanced ETC activity as well as the reduced ATP production.

### 2.6 DCA Promotes the Intracellular Accumulation of Antibiotics and Aggravates the Oxidative Damage of *E. coli*

**Figure 6:**
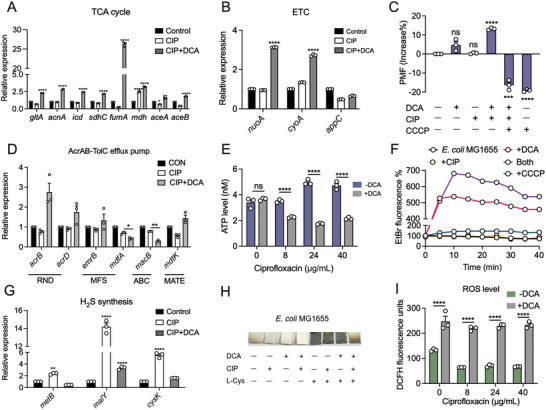
Potentiation mechanisms of DCA to antibiotics against *E. coli*. A,B) The mRNA expression of A) TCA cycle‐related enzymes and B) ETC‐related enzymes in *E. coli* MG1655 under the treatment of CIP (8 µg mL^−1^) with or without DCA (50 × 10^−3^ m). C) PMF changes in *E. coli* MG1655 treated by DCA, CIP, CCCP or their combination, determined by DiOC_2_(3) dye (3 × 10^−3^ m). This dye penetrates and accumulates in the cytosol of bacterial cells when the membrane potential is high. D) Effect of CIP, DCA alone, or their combination on the mRNA expression of efflux pump‐related enzymes in *E. coli* MG1655. E) Intracellular ATP level in *E. coli* MG1655 in the presence of CIP (8 µg mL^−1^) with or without DCA (50 × 10^−3^ m). F) The function of efflux pumps in *E. coli* MG1655 after treatment with CIP (8 µg mL^−1^), DCA (50 × 10^−3^ m) alone, or their combination. CCCP was used as a positive control. G) Effect of CIP with or without DCA (50 × 10^−3^ m) on the mRNA expression of H_2_S synthesis‐related enzymes in *E. coli* MG1655. H) Semiquantitative analysis of H_2_S produced by *E. coli* MG1655 under different treatments after 15 h co‐culture. Representative Pb‐soaked paper strips reflect a brown stain of PbS as a result of the reaction between H_2_S released by bacterial cultures and Pb(Ac)_2_ in the paper strips. I) ROS levels of *E. coli* MG1655 in the presence of increasing concentrations of CIP with or without DCA (50 × 10^−3^ m). All data were determined by unpaired *t*‐test and expressed as mean ± SEM. *p < 0.05, **p < 0.01, ***p < 0.001, ****p < 0.0001. Experiments were performed with biological replicates.

Having shown the modes of action accounting for the direct antibacterial effect of DCA, we next investigated the mechanisms underlying the potentiation of DCA to existing antibiotics. Compared with CIP alone treatment, we found that the combination of DCA and CIP induced the upregulation of genes expression of TCA cycle‐related enzymes as well as ETC‐related enzymes, hinting the increased production of ROS, especially O_2_^•−^ that was released via successive single‐electron reductions in ETC (**Figure** **6**A,B and Figure S4A, Supporting Information).^[^ ^31^ ^]^

It has proven that enough intracellular accumulation is sufficient for antibiotic activity against Gram‐negative bacteria.^[^ ^29b^ ^]^ Thus, we next sought to figure out whether DCA could increase antibiotic accumulation in *E. coli*. To test this, we focused on bacterial proton motive force (PMF) and AcrAB‐TolC efflux pump, which affect antibiotic intake and efflux, respectively. Surprisingly, the combined use of DCA and CIP resulted in elevated PMF compared with CIP treatment. This phenomenon was reversed by the addition of CCCP, a membrane potential disrupter (Figure 6C),^[^ ^32^ ^]^ suggesting that DCA may enhance drug uptake by stimulating the PMF. Considering that intracellular accumulation of antibiotics is the result of the mutual balance between drug intake and drug efflux, thus we further assess the activity of the AcrAB‐TolC efflux pump, which has been documented as the main system responsible for the multiple drug efflux in *E. coli*.^[^ ^33^ ^]^ As expected, the expression level of *mdfA* gene in *E. coli* treated with CIP and DCA, which encodes the major facilitator superfamily transporter MdfA to confer resistance to a number of electroneutral antibiotics,^[^ ^34^ ^]^ was significantly downregulated (Figure 6D). Meanwhile, *macB* gene, encoding ATP binding cassette (ABC)‐type tripartite efflux pump subunit MacB to transport substrates as well as couple with ATP hydrolyzing activity,^[^ ^35^ ^]^ was also dramatically downregulated in the presence of DCA and CIP (Figure 6D). Consistently, the ATP production in the combination treatment of DCA and CIP was significantly reduced compared with that in CIP treatment (Figure 6E). In addition, we used ethidium bromide (EtBr) as a fluorescent probe to holistically evaluate the function of efflux pumps in bacteria after exposure to DCA with or without CIP. As expected, the strengthened intracellular EtBr fluorescence signal was obviously detected in the presence of DCA and CIP group, indicating the depressed function of the efflux pump (Figure 6F). Taken together, these results strongly demonstrate that the addition of DCA not only promotes drug uptake, but also undermines the function of efflux pumps, thereby increasing the intracellular accumulation of antibiotics and their antibacterial activity.

Hydrogen sulfide (H_2_S) has been acknowledged as one of the universal defense systems that protect bacteria against oxidative stress induced by diverse antibiotics.^[^ ^36^ ^]^ Thus, we further investigated whether the combination of DCA and CIP influenced the synthesis of H_2_S. A classic lead acetate reactivity test was used for monitoring the production of H_2_S. Interestingly, the combination treatment resulted in a major deficiency in H_2_S production in *E. coli* MG1655 (Figure 6H). The supplementation of appropriate substrate L‐cysteine in the DCA + CIP group mildly elevated the H_2_S production, whereas remained at a relatively low level compared with other L‐cysteine‐supplied groups, indicating that DCA + CIP indeed blunted the ability to produce H_2_S in bacteria. Consistently, supplying L‐cysteine into the DCA + CIP group aided bacteria to produce more H_2_S and partially scavenged ROS production, ultimately resulting in increased survival (Figure S4B,C, Supporting Information). In agreement with the weakened antioxidant system, ROS level was remarkably increased under the stress of DCA and CIP combination (Figure 6I), resulting in subsequent damage to cells, including DNA, lipids, proteins, etc., and eventually triggering bacterial death.^[^ ^37^ ^]^

Furthermore, we preliminary explored the underlying mechanisms by which the combined use of DCA and CIP remarkably prevents the H_2_S production in *E. coli*. The RT‐qPCR analysis demonstrated that DCA + CIP specifically downregulated the expression levels of several genes including *metB, malY*, and *cysK* (Figure 6G), which all encode different cysteine desulfurases that constitute 3‐mercaptopyruvate sulfurtransferase (3MST), one of the major enzymes that catalyze H_2_S production in *E. coli*.^[^ ^38^ ^]^ However, the in‐depth mechanisms accounting for the negative effect of DCA + CIP on H_2_S‐related gene expression necessitate further studies. Collectively, these data indicate that DCA promotes the accumulation of antibiotics and aggravates the imbalance of oxidant–antioxidant system in *E. coli*, ultimately potentiating the antibacterial activity of existing antibiotics.

## 3 Discussion

Intestinal infections caused by pathogenic bacteria including those with multidrug resistance phenotype pose a global threat to human health.^[^ ^39^ ^]^ According to the World Health Organization (WHO), intestinal infections annually kill more than two million people worldwide, most of whom are children. In some countries, infant mortality caused by intestinal infection is up to 70% of the total mortality of children under 5 years.^[^ ^40^ ^]^ It is of great importance to explore the causative factors accounting for recurrent intestinal infections. Nowadays, SD is implicated in the dysregulation of inflammatory responses and cognitive impairment, chronic negative energy balance, and gradual deterioration of health.^[^ ^41^ ^]^ In the present study, we investigated the impact of SD on the composition of gut microbiota and downstream secondary metabolites, and explored whether these alternations play a critical role in the occurrence of intestinal infections, as well as the potential effects of NMN in this process.

In particular, intestinal health is dynamic and influenced by various environmental factors as well as genetic factors. Thus, understanding the potential relationship between host CR and physiological activities is essential. For example, our previous study reveals that a long‐term high‐fat diet impairs the efficacy of bactericidal antibiotics by altering the composition and diversity of gut microbiota.^[^ ^42^ ^]^ Moreover, a recent study shows that host counteracts infections by nourishing their microbiota with taurine, which boosts the microbiota's production of sulfide and maintains its heightened resistance against subsequent intestinal infections.^[^ ^6^ ^]^ Herein, we demonstrated that SD impairs the CR of intestine in mice whereas NMN supplement rescues it, which provides new insights into the interaction between host activities and intestinal CR, as well as the beneficial roles of NMN on SD‐treated mice.

There is growing evidence that gut microbes are related to host metabolism, homeostatic regulation, and immune system.^[^ ^43^ ^]^ The depletion of specific bacterial taxa, which is induced by the low take of dietary fibers and high take of fat and sugar, could result in gaining risk of chronic inflammatory diseases such as IBD, colorectal cancer, and allergies.^[^ ^44^ ^]^ Our microbial diversity analysis showed that gut microbiota is highly shaped in response to SD while NMN reprograms it. In agreement with our observations, previous studies also reported that sleep restriction, including sleep fragmentation (SF), SD, and circadian rhythm shifts, are linked to gut microbiota dysbiosis and host inflammatory responses.^[^ ^45^ ^]^ For example, chronic SF‐induced alteration of gut microbiota composition was characterized by the preferential growth of highly fermentative members of *Lachnospiraceae* and *Ruminococcaceae* and the decrease of *Lactobacillaceae* families in mice. Moreover, Wang et al. demonstrated that SD reduced several short chain fatty acid‐producing bacteria (e.g., *Alloprevotella*) and decreased circulating butyrate levels, thus aggravating neuroinflammation by ways of lipopolysaccharides‐induced microglial activation and inflammatory responses.^[^ ^46^ ^]^ In contrast, SD‐induced inflammatory responses were attenuated in germ‐free mice. In comparison, the protein level of IL‐6 in our study (Figure S1A, Supporting Information) was higher than that in the previous study.^[^ ^46^ ^]^ It may be due to the fact that 3‐day‐ SD model caused serious damage on the physiological state and intestinal homeostasis, thus resulting in an acute inflammatory response, reflected by the remarkable increase of IL‐6 and decrease of IL‐10 in serum. Consistently, a significant reduction of *α*‐diversity and the obvious difference of *β*‐diversity in SD mice were also observed in these studies.^[^ ^46^, ^47^ ^]^ Notably, our study indicated that the abundance changes of specific *Clostridium* spp. may mediate the decreased CR in SD mice and restored CR after NMN supplementation. Accordingly, most *Clostridium* species are soil or intestinal symbionts or both inhabitants. Only a few are pathogenic microorganisms, examples of which include *Clostridium perfringens*, *Clostridium difficile*, enterotoxigenic *Bacteroides fragilis*.^[^ ^48^ ^]^ Specifically, Clostridium_UCG‐014 was characterized as the beneficial population to support CR as an outcome of producing sufficient DCA. Nevertheless, the biological functions and beneficial roles of *Clostridium* spp. in response to pathogens invasion remain to be explored.

The interaction between gut microbes and host metabolites is multilayered and multicascaded. For example, the alternation of gut microbiota in HFD mice leads to the decrease of the microbiota‐derived metabolites indole‐3‐acetic acid (IAA), thereby impairing bacterial susceptibility to antibiotics.^[^ ^42^ ^]^ Moreover, IAA serves as an endogenous ligand in aryl‐hydrocarbon receptor activation in the cecum and colon epithelium, ultimately reducing proinflammatory factors in piglets.^[^ ^49^ ^]^ In this study, we revealed that reduced microbiota‐derived DCA was responsible for the decreased CR in SD‐treated mice. DCA exhibited potent antibacterial activity as well as a synergistic effect with antibiotics against bacteria, particularly for drug‐resistant pathogens. Consistently, several previous studies also indicated the potential antibacterial activity of DCA against *S. aureus*.^[^ ^7^, ^50^ ^]^ Our mechanistic studies showed that DCA alone enhanced membrane permeability and aggravated oxidative damage, thereby reducing bacteria survival. Furthermore, exogenous DCA promoted antibiotic accumulation and disrupted the balance between oxidative stress and antioxidant defense system, thereby potentiating antibiotic efficacy. Moreover, a previous study demonstrated that isoallolithocholic acid (isoalloLCA) possessing a similar structure to DCA also exerts potential antimicrobial efficacy against Gram‐positive multidrug‐resistant pathogens colonization.^[^ ^51^ ^]^ It is plausible that these two compounds show similar modes of action. However, the specific antibacterial targets of both isoalloLCA and DCA require further investigation.

It is evident that intestinal CR results from a mutualistic, complex, and multilayered interaction between the host, its mucosal immune system, and the commensal microbiota.^[^ ^52^ ^]^ For example, 3‐oxo lithocholic acid (3‐oxoLCA) and isoalloLCA, two distinct derivatives of lithocholic acid (LCA) in the intestinal lumen, directly modulated the balance of T_H_17 and T_reg_ cells to control host immune responses and reduce segmented filamentous bacteria colonization.^[^ ^53^ ^]^ In this study, we did not dwell on the other factors except for the commensal microbiota, but the immune regulation induced by DCA or NMN, in all likelihood, is implicated in intestinal CR and necessitates further studies.

NMN, which acts as an NAD^+^ intermediate, is known for enhancing NAD^+^ biosynthesis.^[^ ^54^ ^]^ A previous study showed that administration of NMN to mice (300 mg kg^−1^) can significantly increase plasma NMN levels within 2.5 min.^[^ ^55^ ^]^ NMN could be transported across the cell membrane by Slc12a8 directly,^[^ ^23^ ^]^ or metabolized to nicotinamide riboside (NR) extracellularly, which is then taken up by the cell and converted to NAD^+^.^[^ ^56^ ^]^ In addition, NMN has the ability to activate SIRT1, which can deacetylate NF‐*κ*B and inhibit its transcriptional activity. Simultaneously, it can reduce the expression of IL‐1, the target of NF‐*κ*B, which may be beneficial to anti‐inflammatory effects.^[^ ^57^ ^]^ Consistently, it was suggested that replenishing NMN reduced the age‐related inflammation of adipose tissue.^[^ ^58^ ^]^ Regarding the safety of NMN, unlike nicotinamide, which causes nausea and induces hepatotoxicity, long‐term (1 year) oral administration of NMN (up to 300 mg kg^−1^) is safe and well‐tolerated for normal wild‐type C57BL/6 mice.^[^ ^55^ ^]^ Recent studies showed that a single oral dose of NMN did not cause any specific deleterious effects in healthy men.^[^ ^59^ ^]^ Combining these results, we reasoned that NMN supplementation maybe a safe regimen to attenuate SD‐induced deletion of CR in mice.

**Figure 7:**
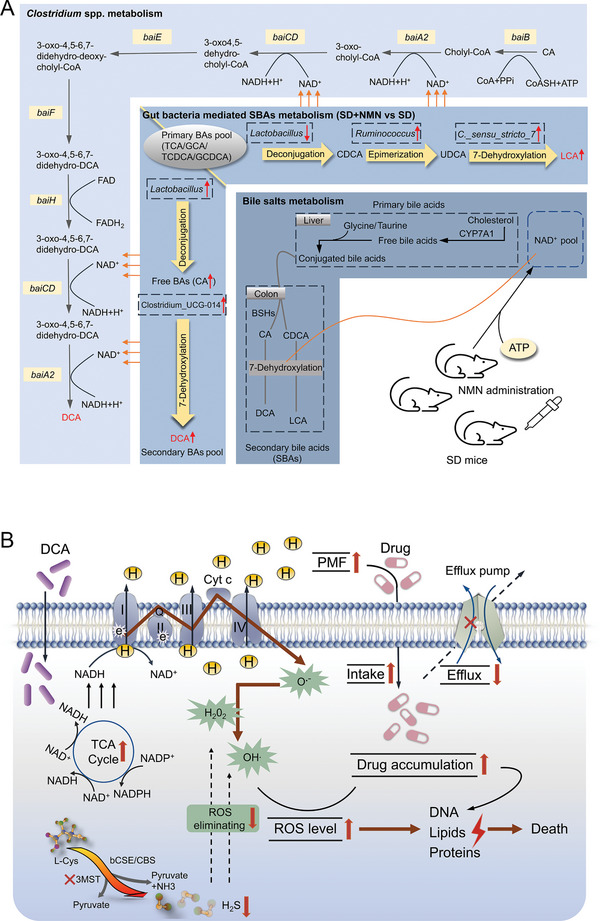
Schematic illustration of the proposed mechanisms for enhanced CR after NMN supplementation in SD mice and the antibacterial actions of DCA. A) The roles of NMN supplementation, gut microbiota, and bile acids metabolism in the maintenance of intestinal CR. In SD mice, decreased *Lactobacillus* reduces deconjugation of primary bile acids, while increased *Ruminococcus* and *C._sensu_stricto_7* accelerate the epimerization of CDCA and the 7‐dehydroxylation of UDCA, thereby promoting the accumulation of LCA (the top right part of the middle layer). In SD + NMN mice, increased *Lactobacillus* and Clostridium_UCG‐014 promote the transformation of primary bile acids to CA and CA to DCA by deconjugation and 7‐dehydroxylation stepwisely, resulting in the increased DCA level (the lower left part of the middle layer). In terms of 7‐dehydroxylation activity in *Clostridium spp*., NMN could positively activate this process by generating NAD^+^. B) Exogenous DCA firstly activates TCA flux and triggers the ETC activity in a NADH‐dependent way, yielding large amounts of superoxide O_2_^•−^ and increasing PMF build‐up by enhanced proton flux. Then, increased PMF promotes drug uptake and impaired efflux pump reduces drug outflow, which aggravates oxidant stress to bacteria cells, and thus inhibits DNA synthesis and replication. Meanwhile, a key enzyme of H_2_S production, 3MST, was inhibited by DCA and CIP, resulting in the compromised ROS elimination efficiency mediated by H_2_S. Overall, exogenous DCA promotes antibiotic accumulation and disrupts the balance between oxidative stress and antioxidant defense system, thereby potentiating antibiotic bactericidal efficacy and facilitating bacterial death. CA, cholic acid; DCA, deoxycholic acid; LCA, lithocholic acid; TCA, taurocholic acid; GCA, glycocholic acid; TCDCA, taurochenodeoxycholic acid; GCDCA, glycochenodeoxycholic acid; CDCA, chenodeoxycholic acid; UDCA, ursodeoxycholic acid; LCA, lithocholic acid; CYP7A1, cytochrome P450 family 7 subfamily A member 1; BSHs, bile acid hydrolases.

In conclusion, we show that SD remarkably impairs intestinal CR in mice, and this defect is correctable with exogenous NMN or DCA supplementation. The impaired CR by SD is correlated with an alternation of gut microbiota, which subsequently causes disorder in the metabolic flux of primary bile acids to secondary bile acids. The increased DCA production in SD + NMN mice not only results from the increased abundance of Clostridium_UCG‐014, but also reaps the benefits from the sufficient NAD^+^ pool in the intestine that is bolstered by NMN administration (**Figure** **7**A). Notably, DCA exerts potent antimicrobial efficacy and potentiates antibiotic therapy against drug‐resistant pathogens both in vitro and in animal models of infection (Figure 7B). Therefore, exploiting the unique bacterial strains‐mediated bile acids biosynthesis pathways to rationally manipulate the bile acids pool and prevent pathogens from invading may represent a reasonable therapeutic alternative against intestinal infections. Overall, our findings not only reveal the negative roles of SD in intestinal CR and the positive roles of DCA or NMN in the process of supporting host CR, but also suggest that the regulation of gut microbiota or bile acids metabolism may serve as a potential strategy to deal with the increasing enteric infectious diseases.

## 4 Experimental Section

### Bacterial Strains and Growth Conditions

All the strains used in this study are shown in Table S1 in the Supporting Information. Bacterial cultures were diluted to 1:100 using fresh Mueller–Hinton broth (MHB, Qingdao Hope Bio‐technology), or M9 minimal media (Na_2_HPO_4_ 6.78 g L^−1^, KH_2_PO4 3.0 g L^−1^, NaCl 0.5 g L^−1^, NH_4_Cl 1.0 g L^−1^, MgSO_4_ 0.241 g L^−1^, CaCl_2_ 0.011 g L^−1^, glucose 4 g L^−1^) and incubated overnight at 37 °C with shaking at 200 rpm. Bacterial cells were centrifuged at 4000 *g* for 7 min at 4 °C, followed by washing two times using sterile saline solution, then cells were suspended in M9 medium to arrive at OD_600_ of 0.5. Harvested cells were used for following biochemical analysis or plate counting. Antibiotics were obtained from the China Institute of Veterinary Drug Control, and other chemical reagents were purchased from Sigma‐Aldrich or TCI Development (Shanghai, China).

### Minimum Inhibitory Concentration (MIC) Assay

MIC values of DCA against a panel of bacterial strains were determined by the standard broth microdilution method, according to the CLSI 2021 guideline.^[^ ^60^ ^]^ In brief, drugs were twofold diluted in MHB and mixed with an equal volume of bacterial suspensions containing ≈1.5 × 10^6^ colony forming units (CFUs) per mL in a clear UV‐sterilized 96‐well microliter plate. After 18 h incubation at 37 °C, the MIC values were defined as the lowest concentration of antibiotics with no visible growth of bacteria. The MICs of DCA alone against pathogens are shown in Table S2 in the Supporting Information.

### Mice Infections

Female BALB/c mice (8 weeks; 25–30 g) were obtained from the Comparative Medicine Center of Yangzhou University and used for all experiments in this study. All animal experiments in this study were performed in accordance with the guidelines of Jiangsu Laboratory Animal Welfare and Ethical of Jiangsu Administrative Committee of Laboratory Animals. The protocols for all animal studies were approved by the Jiangsu Administrative Committee for Laboratory Animals (permission number, SYXKSU‐2007‐0005). The laboratory animal usage license number is SCXK‐2017‐0044, certified by the Jiangsu Association for Science and Technology.

After a week of adaptation, mice were randomly divided into four groups: control group (CON), sleep deprivation group (SD), SD‐NMN‐treated group (SD + NMN), NMN‐treated group (NMN) (*n* = 6 biologically independent animals per group). Consecutive sleep deprivation of mice was performed by a continuously rotating machine (XR‐XS108, XinRuan Information Technology Co., Ltd., Shanghai, China). It is an electrically driven, intelligent instrument, which allowed to set the rotational intensity and period artificially, and permitted the normal diet, for a 30 s pause was appropriate for mice to achieve food and water when needed, and five rotations with alternation of clockwise and counterclockwise implemented the intervene of sleep availably. After 3 days SD, mice were infected with pathogens at a nonlethal dose (1.0 × 10^7^ CFUs) via intragastric administration (i.g.). Then the feces were collected at 12, 24, 48 h post‐infection, serially diluted, and plated onto LB containing selective antibiotics plates to determine the pathogens loads after incubation at 37 °C for 12 h. In SD + NMN group, the mice were administered with a gavage dose of NMN (100 mg kg^−1^) at 10:00 a.m. every day during the SD period. A single intraperitoneal dose of DCA or LCA administration was 100 mg kg^−1^.

### *Galleria mellonella* Infection

The larvae of *Galleria mellonella* (Huiyude Biotech Company, Tianjin, China) were randomly divided into two groups (*n* = 10 per group, control group and treated group) and infected with 10 µL of *E. coli* B2 or MRSA T144 suspension (1.0 × 10^8^ CFUs) at the right posterior gastropoda. After 1 h postinfection, all larvae were treated with CIP (10 × 10^−6^ m kg^−1^) at the left posterior gastropoda. 30 min later, the treated group was injected with a single dose of DCA at 20 × 10^−6^ m kg^−1^, then the survival rate was monitored for 5 days. In the single DCA treatment model, the larvae were injected with *E. coli* B2 or MRSA T144 suspension (1.0 × 10^5^ CFUs) and coincident DCA supplement (10 × 10^−6^ m kg^−1^).

### Histological Procedures

Samples from the colon were embedded in O.C.T. (Sakura), snap‐frozen in liquid nitrogen, and stored at −80 °C. Sections (5 µm) were mounted on glass slides, air‐dried at room temperature for 2 h, and stained with hematoxylin and eosin (H&E). H&E pictures were taken by optical microscope (Leica 1100). An automatic best fit was applied for the best contrast.

### Enzyme‐Linked Immunosorbent Assay (ELISA)

Treated mice were euthanized for collection of serum samples after 3 days sleep deprivation. The fresh blood samples were stored at room temperature for 4–6 h and then stored at 4 °C for 12 h, the well‐precipitated upper serum samples were soaked up carefully for the following ELISA. Cytokines (IL‐6, IL‐1*β*, TNF‐*α*, IL‐4, IL‐10, and INF‐*γ*) were detected using commercial ELISA kits in accordance with the manufacturer's protocol. ELISA kits for cytokines determination were purchased from Beyotime (Shanghai, China).

### Determination of Pathogens Colonization in the Gut

Fecal pellets were collected directly from mice with a sterile 1.5 mL tube. The weight of each fecal sample was recorded. Then fecal were supplied with phosphate‐buffered saline (PBS, 1 mL) and ground by ultrasonic shocker (Shanghai Jingxin Tissuelyser‐32). 50 µL stool suspension was serially tenfold diluted in sterile saline and spot‐plated onto LB agar plates containing the selective antibiotics.

### Analysis of Microbiota Composition

Gut microbiota profiling of fecal samples from CON, SD, SD + NMN, and NMN mice after 3 days sleep deprivation was performed by Majorbio Bio‐Pharm Technology (Shanghai, China). Fresh fecal samples were collected and DNA extraction was performed using the E.Z.N.A stool DNA kit. The amplicons of the V3–V4 region within the 16S rRNA gene were sequenced on the Illumina MiSeq platform. Optimized primers (338F, 5′‐ACTCCTACGGGAGGCAGCAG‐3′; 806R, 5′‐GGACTACHVGGGTWTCTAAT‐3′). These sequences were filtered using Mothur (v.1.35) and then binned into OTUs. Taxonomic assignments were determined using a naive Bayesian classifier with the Ribosomal Database Project (RDP) training set (v.11; http://rdp.cme.msu.edu/) requiring a 70% bootstrap confidence score.

### Untargeted Metabolomics Analyses

Fecal samples (about 50 mg) were collected from CON, SD, SD + NMN, and NMN‐treated mice after 3 days of sleep deprivation. The metabolite extracts from mice fecal samples were analyzed using a UPLC‐Q‐Exactive Orbitrap mass spectrometer (Thermo Fisher Scientific). Samples were subjected to LC for component separation, single component re‐entry to the ion source of the high vacuum mass spectrometer for ionization, separated by mass to charge ratio (*M*/*Z*) to obtain mass spectra (50–750 *m/z*), then the mass spectrometric data of the samples were analyzed to obtain qualitative and quantitative results. Each sample was analyzed by ultra‐performance liquid chromatography‐mass spectrometry in both positive and negative ionization modes. Metabolites were characterized via comparison of the retention times, change/mass ratio values, and fragmentation patterns with those previously reported.^[^ ^61^ ^]^ The internal standards were used to ensure the consistency between chromatography and injection. Instruments were tuned and calibrated for mass resolution and mass accuracy daily.

### Determination of DCA Sensitivity across Individual Microbiota Strains

All strains were grown overnight at 37 °C, diluted in fresh MH broth (1:1000), and added into a 96‐well plate in triplicate. DCA ranged from 0 × 10^−3^ to 125 × 10^−3^ m was added to cultures by serial dilution. OD_600_ values were determined by each hour within 8 h, and the antimicrobial efficiency of DCA at 6 h was presented.

### Survival Assays

Overnight bacterial cells were diluted 1:100 in MH broth and grown to exponential phase, washed, and resuspended in M9 minimal media. Then a mixture of bacteria and DCA (final concentrations, 0 × 10^−3^ to 80 × 10^−3^ m) or antibiotics alone or both were incubated at 37 °C for 6 h. Percent survival was performed by serially diluted and spot‐plated onto LB agar plates. The percentage of survival was determined by dividing the CFUs obtained from a treated sample by the CFUs obtained from control.

### Growth Curve Assays

Growth curves were obtained based on the automated growth analysis system in the Infinite M200 Microplate reader (Tecan). *E. coli* MG1655 was grown overnight at 37 °C, diluted in fresh MH broth (1:1000), and added into a 96‐well plate in triplicate. Then DCA (final concentration, 50 × 10^−3^ m) or appropriate antibiotics as described in the text or figure legends were added to the 96‐well plate. The total volume was up to 200 µL. OD_600_ values were recorded automatically within 10 h and the means of the triplicate cultures were plotted.

### RT‐qPCR Analysis

RNA extraction was carried out as previously described.^[^ ^62^ ^]^ Total RNA of *E. coli* B2 treated with DCA, CIP, or their combination was isolated using the EASYspin Plus kit (Aidlab, Beijing, China) and quantified by the ratio of absorbance (260 nm/280 nm) using a Nanodrop spectrophotometer (Thermo Scientific, MA, USA). The first‐strand cDNA from all bacterial cells was synthesized using the PrimeScript RT reagent Kit with gDNA Eraser (Vazyme, Nanjing, China) following the manufacturer's protocols. Thermal cycling was performed by a two‐step PCR amplification standard procedure with 95 °C for 30 s and 40 cycles of 95 °C for 5 s, 60 °C for 34 s. RT‐qPCR test was performed using a 7500 Fast Real‐Time PCR System (Applied Biosystem, CA, USA). The fold changes of gene expression were determined using the 2^−ΔΔCt^ method. Primer sequences used in this study are listed in Table S3 in the Supporting Information.

### Total ROS Determination

Total ROS level in *E. coli*, probed by 2′,7′‐dichlorodihydrofluorescein diacetate (DCFH‐DA, 10 × 10^−6^ m), was determined by the Infinite M200 Microplate reader (Tecan) with the excitation wavelength of 488 nm and emission wavelength of 525 nm. Briefly, the overnight cells were diluted at 1:100 and grown to an exponential phase, washed, and suspended in M9 media. Cells were adhered to DCFH‐DA and co‐incubated for 1 h, washed in PBS for three times. Then cultures were mixed with varying concentrations of DCA (final concentrations, 0 × 10^−3^ to 80 × 10^−3^ m), CIP (final concentrations, 0 to 40 µg mL^−1^), or their combination, then incubated at room temperature for 1 h.

### Membrane Permeability

The membrane permeability of bacteria cells was determined using fluorescent probe propidium iodide (PI, 10 × 10^−9^ m). The fluorescent intensity of PI‐probed cells in the presence of DCA (50 × 10^−3^ m) or increasing CIP (final concentrations, 0 to 40 µg mL^−1^) or their combination was measured with the excitation wavelength at 535 nm and emission wavelength at 615 nm.

### NAD^+^/NADH Determination

The overnight culture was washed and resuspended with M9 media to obtain an OD_600_ of 0.5. After treatment with different concentrations of DCA (final concentrations, 0 × 10^−3^ to 80 × 10^−3^ m), and CIP (final concentrations, 0 to 40 µg mL^−1^) alone or the combination with DCA at a final concentration of 50 × 10^−3^ m for 4 h, then cell pellets were washed and re‐suspended with 200 µL precooled extraction buffer. After centrifuging at 12 000 *g* for 10 min at 4 °C, the supernatant was divided into two equal volumes (90 µL), one was used for the determination of the total amount of NAD^+^ and NADH and the other was used for detecting the amount of NADH only. All steps were followed by the protocols of the NAD^+^/NADH Assay Kit with WST‐8 (Beyotime, Shanghai).

### Intracellular ATP Level Measurement

The pretreatment of *E. coli* B2 used for the ATP measurement was similar to that of the NAD^+^/NADH determination. After treatment with different concentrations of DCA, and CIP alone or in combination with DCA at a final concentration of 50 × 10^−3^ m for 4 h, bacterial cultures were centrifuged at 12 000 *g* at 4 °C for 7 min, and the supernatant was removed. Bacterial cells were lysed by precooled lysozyme, centrifuged, and the supernatant was collected for determining the intracellular ATP levels. The detecting solution was added to a 96‐well plate and incubated at room temperature for 5 min. The supernatants were then added to the wells and mixed fleetly with ATP detection working solution, then the luminescence signals were determined by the Infinite M200 Microplate reader (Tecan). All steps were performed based on the manufacturer's protocols for ATP determination (S0027, Beyotime).

### PMF Determination

Bac*Light* bacterial membrane potential kit (Thermo Fisher Scientific) was used to measure the PMF. Briefly, 1 mL bacterial culture obtained from the log‐phase was directly diluted to 10^6^ CFUs per mL in the culture medium without washing and co‐cultured with 10 µL of 3 × 10^−3^ m DiOC_2_(3) (3,3’‐diethyloxa‐carbocyanine iodide) as treated samples. Another two tubes should be prepared for a depolarized control and an unstrained control. 10 µL of 500 × 10^−6^ m CCCP was added to the depolarized control sample and mixed. Samples were incubated at 37 °C for 30 min. Signal intensity was assessed by CytExpert Flow Cytometer (Beckman, USA) and analyzed by FlowJo V10.8.1. The green fluorescence was detected through a 488 to 530 nm bandwidth band‐pass filter, and the red fluorescence was detected through a 488 to 610 nm bandwidth band‐pass filter. The forward scatter, side scatter, and fluorescence were collected with logarithmic signal amplification. The PMF was determined and normalized as the intensity ratio of the red/green fluorescence. The membrane potential was calculated with the following formula: PMF = lg(10^3/2^×($\frac{\mathrm{red}\;\mathrm{fluorescence}}{\mathrm{green}\;\mathrm{fluorescence}}))$.

### Efflux Pump Determination

The effect of DCA or CIP or their combined use on the function of efflux pump was evaluated using EtBr efflux assay according to previous study.^[^ ^62^ ^]^ Cells were co‐incubated with EtBr (final concentration, 5 × 10^−6^ m) and DCA, CIP, both, or known efflux pump inhibitor CCCP (0.1 × 10^−3^ m) at 37 °C to an OD_600_ of 0.5. After centrifuging at 4000 *g* at 4 °C for 7 min, the pellets were collected and resuspended in fresh M9 media. Subsequently, fluorescence intensity was monitored with the excitation wavelength at 530 nm and emission wavelength at 600 nm during 40 min.

### H_2_S Detection

A lead acetate detection method was utilized to monitor H_2_S production in *E. coli* MG1655. Overnight cultures were diluted 1:100 in LB with or without L‐Cys (final concentration, 20 × 10^−3^ m) and incubated for 15 h at 37 °C with Pb(Ac)_2_ paper strips affixed to the inner wall of a cultural tube. According to the following reaction: Pb(Ac)_2_ + H_2_S = PbS + 2HAc, the darker the paper strip was, the more H_2_S was produced.

### Statistical Analysis

Statistical analysis was performed using GraphPad Prism version 9.0 and SPSS software. Data were presented as mean ± SEM. For the in vitro studies, unpaired *t*‐test (normally distributed data) between two groups or one‐way analysis of variance (ANOVA) among multiple groups was used to calculate *p*‐values. For the in vivo studies, data between two groups were analyzed by unpaired *t*‐test if the data exhibited a Gaussian distribution and equal variance, or by unpaired *t‐*test with Welch's correction. Differences with *p* < 0.05 were considered significant. Significance levels were indicated by numbers of asterisks: *p < 0.05, **p < 0.01, ***p < 0.001. The data of metabolite profiles and microbiota profiles were analyzed on the online platform of Majorbio Cloud Platform (https://cloud.majorbio.com/).

## Conflict of Interest

The authors declare no conflict of interest.

## Author Contributions

Y.L. and Z.W. designed and conceived the project. D.F., T.X., J.S., J.S., F.L., and Y.Y. performed all experiments. Y.L., D.F., and T.X. analyzed the data and prepared all figures. Y.L. and D.F. wrote the manuscript. All authors reviewed the manuscript.

## Supporting information

Supporting Information

## Data Availability

The data that support the findings of this study are available from the corresponding author upon reasonable request.
