# Supplementary material for: Nicotinamide Mononucleotide Ameliorates Sleep Deprivation‐Induced Gut Microbiota Dysbiosis and Restores Colonization Resistance against Intestinal Infections
Source: Adv Sci (Weinh). 2023 Jan 25;10(9):2207170. doi: 10.1002/advs.202207170 (PMC10037695; doi:10.1002/advs.202207170)
Supplement: Supplementary file 1 — Supporting Information [file ADVS-10-2207170-s001.pdf]

ADVANCED SCIENCE
Open Access

Supporting Information

for *Adv. Sci.*, DOI 10.1002/advs.202207170

Nicotinamide Mononucleotide Ameliorates Sleep Deprivation-Induced Gut Microbiota Dysbiosis and Restores Colonization Resistance against Intestinal Infections

Dan Fang, Tianqi Xu, Jingyi Sun, Jingru Shi, Fulei Li, Yanqing Yin, Zhiqiang Wang and Yuan Liu**

Supporting Information

Nicotinamide mononucleotide ameliorates sleep deprivation-induced gut microbiota dysbiosis and restores colonization resistance against intestinal infections

Dan Fang[1], Tianqi Xu[1], Jingyi Sun[1], Jingru Shi[1], Fulei Li[1], Yanqing Yin[1],
Zhiqiang Wang[1,2,3]*, Yuan Liu[1,2,3]*

[1]College of Veterinary Medicine, Yangzhou University, Yangzhou 225009, China

[2]Jiangsu Co-innovation Center for Prevention and Control of Important Animal Infectious Diseases and Zoonoses, Joint International Research Laboratory of Agriculture and Agri-Product Safety, the Ministry of Education of China, Yangzhou University, Yangzhou 225009, China

[3]Institute of Comparative Medicine, Yangzhou University, Yangzhou 225009, China

***Corresponding authors:** zqwang@yzu.edu.cn (Z.W.) and liuyuan2018@yzu.edu.cn (Y.L.)

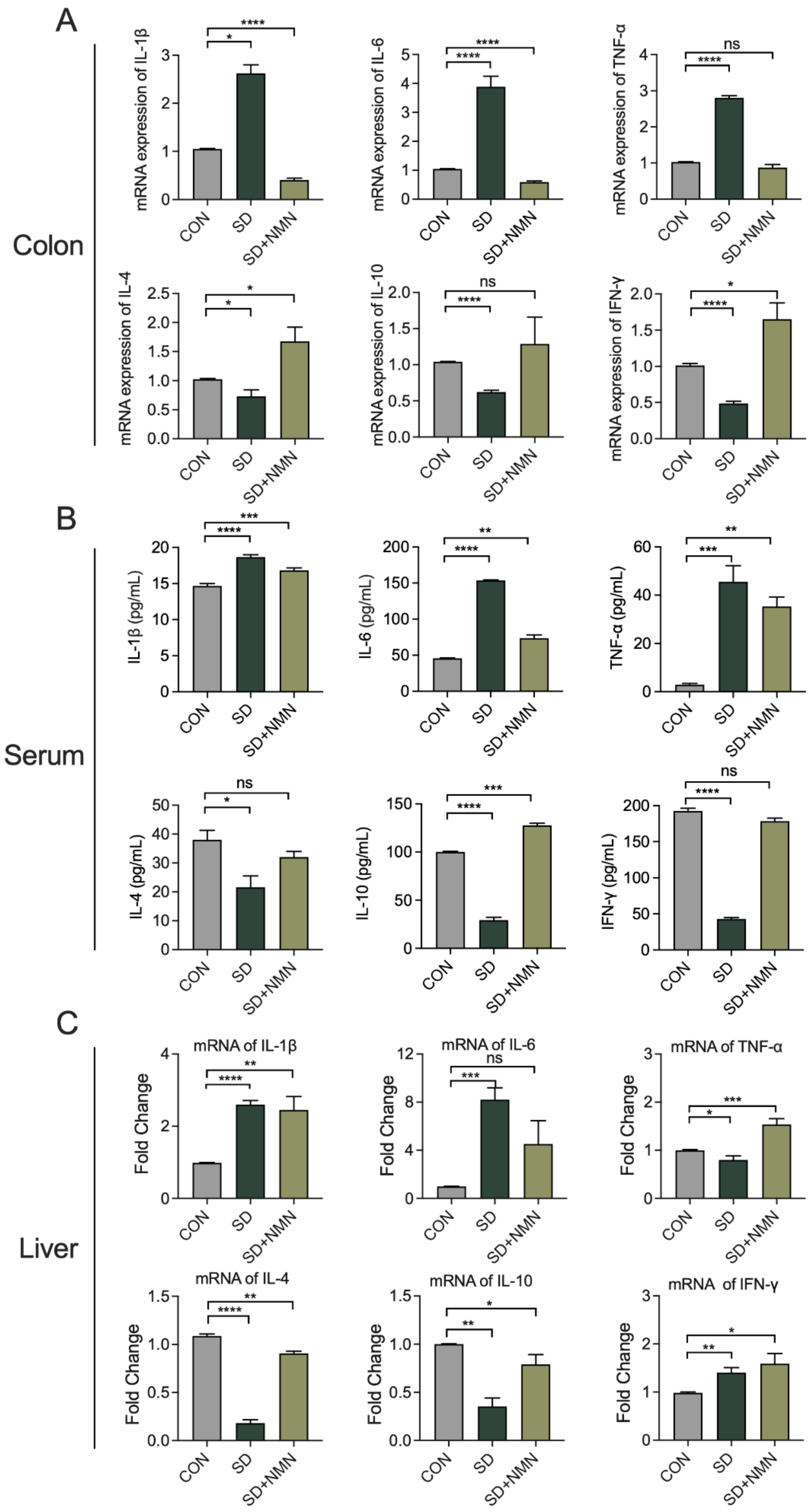

A
Colon
mRNA expression of IL-1β
mRNA expression of IL-6
mRNA expression of TNF-α
mRNA expression of IL-4
mRNA expression of IL-10
mRNA expression of IFN-γ
B
Serum
IL-1β (pg/mL)
IL-6 (pg/mL)
TNF-α (pg/mL)
IL-4 (pg/mL)
IL-10 (pg/mL)
IFN-γ (pg/mL)
C
Liver
mRNA of IL-1β
mRNA of IL-6
mRNA of TNF-α
mRNA of IL-4
mRNA of IL-10
mRNA of IFN-γ
Fold Change
CON
SD
SD+NMN
ns

Figure S1. mRNA expression of inflammatory cytokines in the mice.

(A) The mRNA expression of inflammatory cytokines in the colon of mice, including pro-inflammatory cytokines (IL-1β, IL-6, TNF-α) and anti-inflammatory cytokines (IL-4, IL-10 and INF-γ). mRNA abundances were determined by RT-qPCR and relative gene expressions were normalized with β-actin.

(B) The protein abundance of pro-inflammatory cytokines (IL-1β, IL-6, TNF-α) and anti-inflammatory cytokines (IL-4, IL-10 and INF-γ) in the serum from CON, SD and SD + NMN groups respectively. Data were representative of three independent experiments with three replicates per group.

(C) Fold changes of mRNA expression of inflammatory cytokines in the livers of mice, including pro-inflammatory cytokines (IL-1β, IL-6, TNF-α) and anti-inflammatory cytokines (IL-4, IL-10 and INF-γ). mRNA abundances were determined by RT-qPCR and relative gene expressions were normalized with β-actin.

Data are presented as mean ± SEM and statistical significance are identified by Student's *t*-test. Experiments were performed with biological replicates.

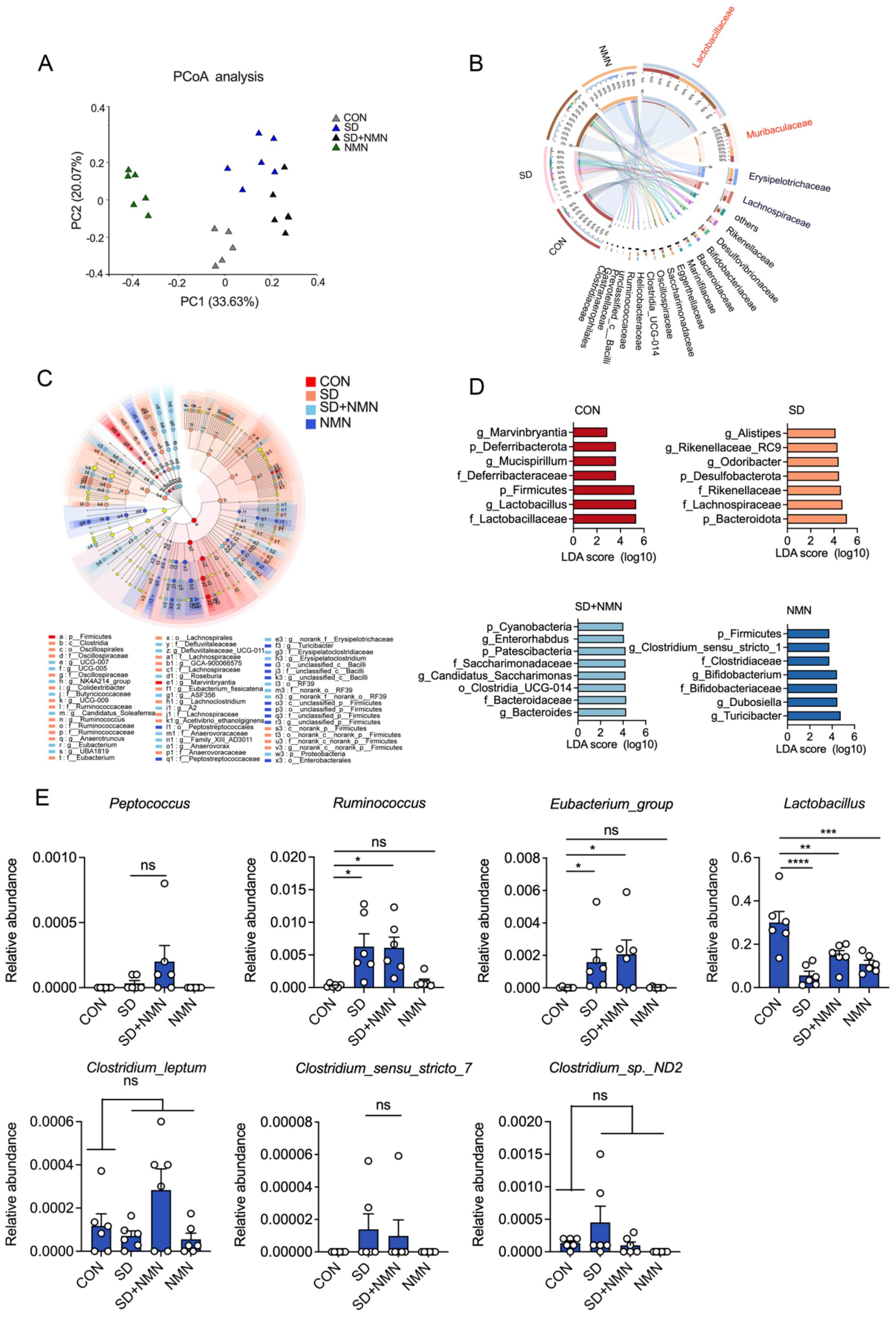

Figure S2. Microbial diversity analysis of altered gut microbiota among four groups.

(A) Principal component analysis (PCA) plot of CON, SD, SD + NMN and NMN samples along principal component (PC) 1 and 2, which explained 52.1% and 20.07% of the total variance, respectively.

(B) Shown are comparisons of the dominant bacterial population at the class level for each group. The width of the bars from each class indicates the relative abundance of that class in each group.

(C) Cladogram generated by LEfSe analysis showing enriched taxa in four groups, including CON (red), SD (orange), SD + NMN (light blue) and NMN (dark blue).

(D) LDA scores of enriched taxa from **C** (LDA>2).

(E) Bile acids metabolism-related gut microbiota abundance in four groups.

Data in **(E)** were displayed as mean ± SEM, and statistical significance were determined by unpaired *t*-test (*$P < 0.05$; **$P < 0.01$; ***$P < 0.001$; ***$P < 0.001$; ns, not significant).

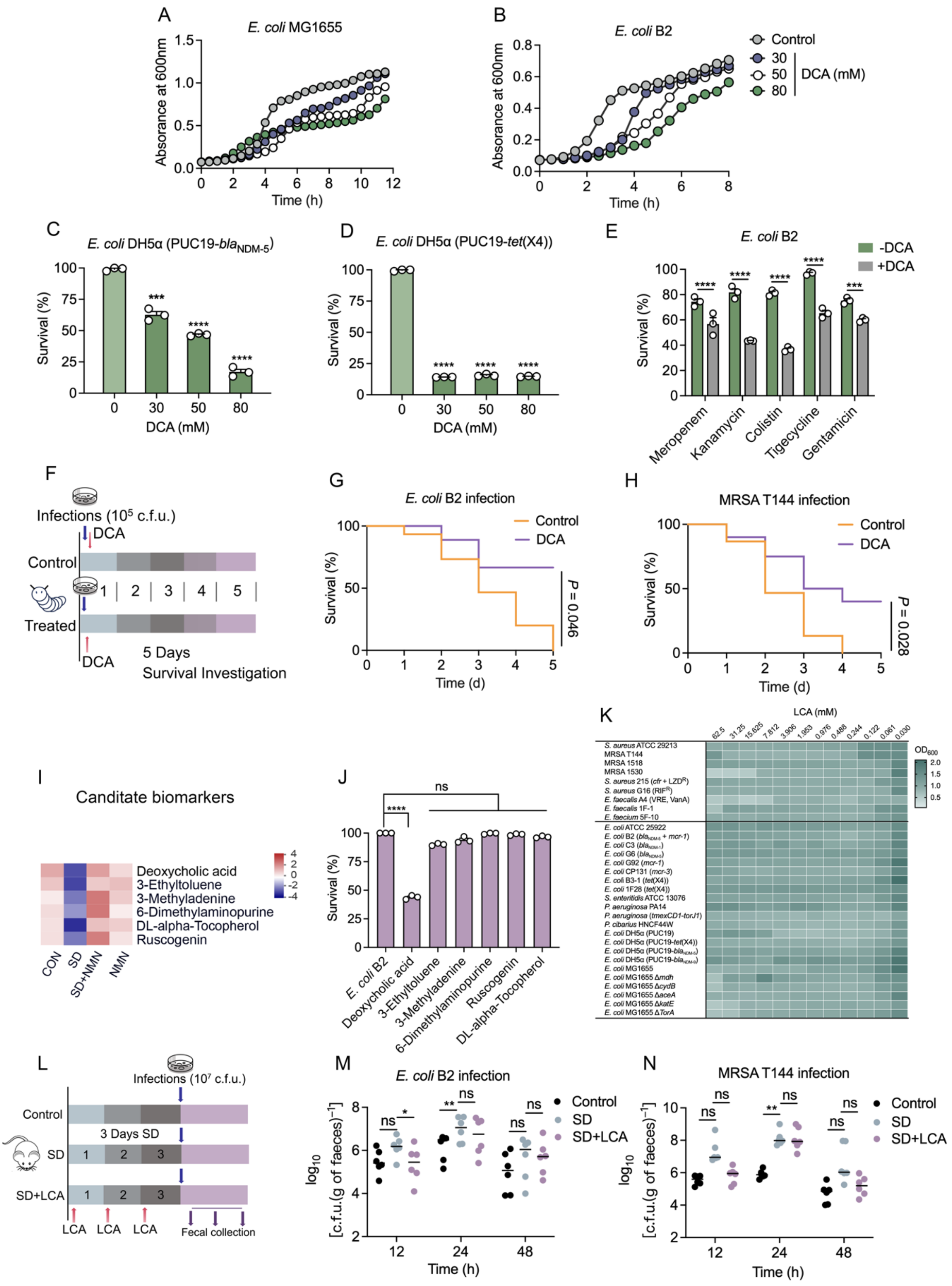

Figure S3. Antimicrobial activity analysis of DCA and LCA.

(A and B) Representative growth curves of *E. coli* MG1655 and *E. coli* B2 in the presence of varying concentrations of DCA. The curves represent averaged values with a <5% margin of error.

(C and D) Percent survival of engineered *E. coli* DH5α (PUC19-*bla*$_{NDM-5}$) and *E. coli* DH5α (PUC19-*tet*(X4)) treated by increasing concentrations of DCA.

(E) DCA exhibits the synergistic effect with different classes of antibiotics, including meropenem, kanamycin, colistin, tigecycline and gentamicin, against *E. coli* B2.

(F) Protocol of the therapeutic potential assessment of single DCA administration in larvae.

(G and H) Monotherapy of DCA (20 μM/kg) improves the survival rate of *Galleria mellonella* larvae after 5 days post-infection by *E. coli* B2 or MRSA T144 (10^5 CFUs per larvae).

(I) The potential biomarkers that decreased in the SD group but elevated in the SD+NMN group were presented in the form of a heatmap. Bule to red colors correspond to low to high abundance.

(J) Evaluation of the antimicrobial effect of the candidate metabolites against *E. coli* B2 and only DCA (50 mM) exhibits significant antibacterial properties.

(K) LCA sensitivity analysis in response to individual microbiota strains, including a panel of clinically drug-resistant strains. The value of OD_{600} can be compared relative to a light-green and dark-green gradient. Light green has the lowest value and dark green has the highest value.

(L) Experimental protocols of LCA-supplied intestinal infection model. Three-days LCA administration: 100 mg/kg/day (*i.p.*).

(M and N) Bacterial loads in the feces of mice at 12, 24, 48 h post infection by *E. coli* B2 **(M)** or MRSA T144 **(N)**.

Values are presented as mean ± SEM. Statistical significance was determined by unpaired *t*-test and denoted as follows: *$P < 0.05$; **$P < 0.01$; ***$P < 0.001$; ns, not significant.

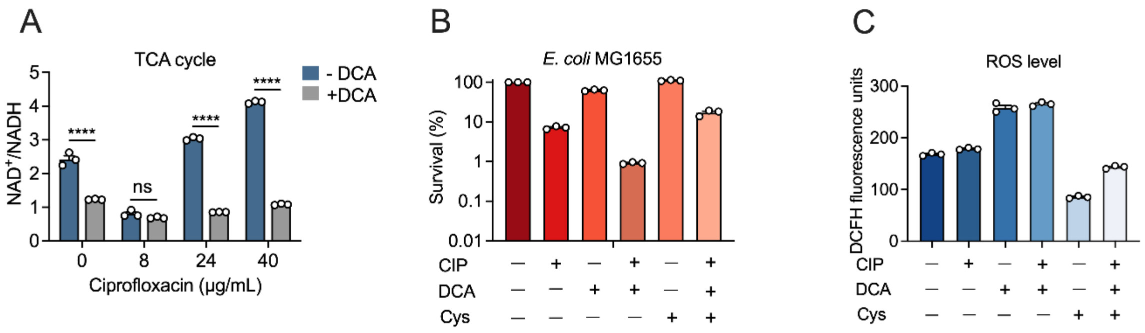

Figure S4. Biochemical factors determination of *E. coli* MG1655 in the presence of DCA and CIP.

(A) Intracellular NAD^+ /NADH ratio in the presence of CIP with or without DCA.

(B) Comparison of *E. coli* MG1655 survival in the presence of combination treatment with or without L-cys.

(C) Comparison of ROS levels in *E. coli* MG1655 in the presence of combination treatment with or without L-cys.

Values are presented as mean ± SEM. Statistical significance was determined by unpaired *t*-test and denoted as follows: *$P < 0.05$; **$P < 0.01$; ***$P < 0.001$; ns, not significant.

Supplementary Tables

Supplementary Table 1 Bacterial strains used in this study.

Organism and phenotypes	Sources/references
**Gram-positive bacteria**	
S. aureus ATCC 29213	ATCC
MRSA T144	[1]
MRSA 1518	[1]
MRSA 1530	In this study
S. aureus 215 (*cfr* + LZD^R)	[1]
S. aureus G16 (RIF^R)	[1]
E. faecalis A4 (*tet*(A), *van*A, Van^R)	[2]
E. faecalis 1F-1	[2]
E. faecium 5F-10	[2]
**Gram-negative bacteria**	
E. coli ATCC 25922	ATCC
E. coli B2 (bla_{NDM-5} + *mcr-1*)	[3]
E. coli C3 (bla_{NDM-1})	[4]
E. coli G6 (bla_{NDM-5})	[4]
E. coli G92 (*mcr-1*)	In this study
E. coli CP131 (*mcr-3*)	In this study
E. coli B3-1 (*tet*(X4))	[4]
E. coli 1F28 (*tet*(X4))	In this study
S. enteritidis ATCC 13076	ATCC
P. aeruginosa PA14	In this study
P. aeruginosa (VIM + *tmexCD1-torJ1*)	[5]
P. cibarius HNCF44W (bla_{NDM-1}+ *tet*(X6))	[6]
E. coli DH5α (PUC19)	In this study
E. coli DH5α (PUC19-*tet*(X4))	In this study
E. coli DH5α (PUC19-bla_{NDM-5})	In this study
E. coli DH5α (PUC19-bla_{NDM-9})	In this study
E. coli MG1655	[7]
E. coli MG1655△*mdh*	[7]
E. coli MG1655△*cydB*	[7]
E. coli MG1655△*aceA*	[7]
E. coli MG1655△*katE*	[7]
E. coli MG1655△*torA*	[7]

ATCC, American type culture collection; MRSA, Methicillin-resistant *staphylococcus aureus*; LZD^R, linezolid resistance; Van^R, vancomycin resistance;

References

[1] Y. Liu, S. Ding, R. Dietrich, E. Märtlbauer, K. Zhu, *Angew. Chem. Int. Ed.* **2017**, *56*, 1486.

[2] Y. Liu, Y. Jia, K. Yang, R. Li, X. Xiao, Z. Wang, *ACS Infect. Dis.* **2020**, *6*, 761.

[3] M. Song, Y. Liu, X. Huang, S. Ding, Y. Wang, J. Shen, K. Zhu, *Nat. Microbiol.* **2020**, *5*, 1040.

[4] Y. Liu, Y. Jia, K. Yang, R. Li, X. Xiao, Z. Wang, *Commun. Biol.* **2020**, *3*, 162.

[5] Q. Wang, K. Peng, Y. Liu, X. Xiao, Z. Wang, R. Li, *Antimicrob. Agents Chemother.* **2021**, *65*, e0271220.

[6] Y. Li, Q. Wang, K. Peng, Y. Liu, R. Li, Z. Wang, *Front. Microbiol.* **2020**, *11*, 1940.

[7] Y. Liu, Y. Jia, K. Yang, Z. Tong, J. Shi, R. Li, X. Xiao, W. Ren, R. Hardeland, R. J. Reiter, Z. Wang, *Theranostics* **2020**, *10*, 10697.

Supplementary Table 2 Antimicrobial spectrum of DCA against bacterial strains.

Strains	DCA (MIC, mM)
**Gram-positive bacteria**	
S. aureus ATCC 29213	0.97
MRSA T144	0.97
**Gram-negative bacteria**	
E. coli ATCC 25922	125
E. coli DH5α (PUC19-*tet*(X4))	125
E. coli DH5α (PUC19-bla_{NDM-5})	125
E. coli DH5α (PUC19-bla_{NDM-9})	125

Supplementary Table 3 RT-qPCR primers used in this study.

Genes	Sequence (5'→3')
acrB-F	AAGAAGCTACCCGTAAGTCG
acrB-R	AGTAGAACCGCCAAAGAAGG
acrD-F	TGGAATCGTTAGTGAAGCAG
acrD-R	CAGCCAGACACAGGAATAC
emrB-F	TCTCATTGGCGGAAATAATCAG
emrB-R	TAAACCCGTTCAACCCGAAT
mdfA-F	TTGATTGGGTTCCTACTTCG
mdfA-R	CCAGACAGGTGACGATAAAC
macB-F	GGCGTCTTGAAAACTGTTGA
macB-R	GTCACTGACACCAGCATAATA
mdtK-F	TAATGTTCGTGCTTCCAATG
mdtK-R	CATACAGACACCCACCATAA
gltA-F	CACCCTCAACGGGGATACAG
gltA-R	GATGCGGTTGAAGTGAAGCC
acnA-F	CGTATCTCGACGAACTGGGG
acnA-R	CGTTTCGATAGGATCGGGCA
icd-F	CCCGAACACTGGCAAAGAGA
icd-R	GCCAGGGCGTCAGAAATGTA
sdhC-F	GCAGTTTGCTCACGTCAAGG
sdhC-R	TCACGGAAATGATGCTGGCT
fumA-F	CCTTACCGCTCTGGCGTATC
fumA-R	TGCGAGAAGTGAAAGCACGA
mdh-F	TACTTCGCTCACGACATCCG
mdh-R	CCTGACGGTTGATCTTCGCT
aceA-F	CGGTTATTGGCGGTCACTCT
aceA-R	CGTTCTGGATGCGTTTGGTC
aceB-F	CGCGAAATATCCGGGCAAAC
aceB-R	GCCAGGGTGATGAACTGGAA
nuoA-F	AGTCATCGCTCATCACTGGG
nuoA-R	CGACCGCCTAAAAACCAACC
cyoA-F	CCGAACTGGTCACACTCCAA
cyoA-R	TGGTAATGGGCTTCTCGTCG
appC-F	TCGAATCCACCTTTGTCGGG
appC-R	GATTTGAACCGAACGCCACC
metB-F	GTCGTTGCCGGAAAATCAGG
metB-R	GCCAGCGTAAACAACGACAG
malY-F	AAGCAGGCTGATGGTTGGTT
malY-R	CATCGCACGTCCACACTTTC

cysK-F	TTATTGCTGGCGTTGGGACT
cysK-R	GCTCAACGGCGACAGAGATA
IL-1β-F	ATGAAAGACGGCACACCCAC
IL-1β-R	GCTTGTGCTCTGCTTGTGAG
TNF-α-F	AGGCACTCCCCCAAAAGAT
TNF-α-R	TGAGGGTCTGGGCCATAGAA
IFN-γ-F	GCTTTGCAGCTCTTCCTCA
IFN-γ-R	CTTTTGCCAGTTCCTCCAG
IL-6-F	TGCAAGAGACTTCCATCCAGT
IL-6-R	GTGAAGTAGGGAAGGCCG
IL-4-F	CCATATCCACGGATGCGACA
IL-4-R	AAGCACCTTGGAAGCCCTAC
IL-10-F	GGCGCTGTCATCGATTTCTC
IL-10-R	ATGGCCTTGTAGACACCTTGG
β-actin-F	GTCCACCTTCCAGCAGATGT
β-actin-R	GAAAGGGTGTAAAACGCAGC
